# Cell Specific CD44 Expression in Breast Cancer Requires the Interaction of AP-1 and NFκB with a Novel *cis*-Element

**DOI:** 10.1371/journal.pone.0050867

**Published:** 2012-11-30

**Authors:** Shannon M. Smith, Li Cai

**Affiliations:** 1 Department of Cell and Developmental Biology, Rutgers University, Piscataway, New Jersey, United States of America; 2 Department of Biomedical Engineering, Rutgers University, Piscataway, New Jersey, United States of America; National University of Ireland Galway, Ireland

## Abstract

Breast cancers contain a heterogeneous population of cells with a small percentage that possess properties similar to those found in stem cells. One of the widely accepted markers of breast cancer stem cells (BCSCs) is the cell surface marker CD44. As a glycoprotein, CD44 is involved in many cellular processes including cell adhesion, migration and proliferation, making it pro-oncogenic by nature. CD44 expression is highly up-regulated in BCSCs, and has been implicated in tumorigenesis and metastasis. However, the genetic mechanism that leads to a high level of CD44 expression in breast cancer cells and BCSCs is not well understood. Here, we identify a novel *cis*-element of the CD44 directs gene expression in breast cancer cells in a cell type specific manner. We have further identified key *trans-acting* factor binding sites and nuclear factors AP-1 and NFκB that are involved in the regulation of cell-specific CD44 expression. These findings provide new insight into the complex regulatory mechanism of CD44 expression, which may help identify more effective therapeutic targets against the breast cancer stem cells and metastatic tumors.

## Introduction

Breast cancer remains the most common form of cancer among women and the second leading cause of cancer related deaths [1]. Recently a small subset of cancer cells was identified by their cell surface markers (e.g., up-regulation of CD44 and down-regulation of CD24) as cancer stem cells (CSCs) [2]. This CD44^+^/CD24^low/−^ signature is observed in other CSCs including prostate, pancreatic, brain and leukemia stem cells [3]–[5]. In addition to stem cell characteristics (i.e., the ability to self-renew and differentiate into all cell types in a mammary gland), CSCs are resistant to chemo- and radiation treatment [6], and have the increased ability to metastasize and develop new tumors throughout the body [7].

As a cell surface glycoprotein, CD44 is ubiquitously expressed on most cells throughout the body [8]–[10]. CD44 is involved in cellular processes including cell-cell and cell-extracellular matrix adhesion, migration, differentiation and survival, all of which makes CD44 pro-oncogenic by nature [9], [11]–[13]. Studies have established that CD44 is a therapeutic target for metastastic tumors [14]. By targeting CD44, human acute myeloid leukemic stem cells can be eradicated [5]. In addition, directly repressing CD44 expression by miR-34a inhibits prostate CSCs and metastasis [15].

Overexpression of CD44 has been correlated to a number of transcription factors including Egr1, AP-1, NFκB, and c/EBPβ [8]. Most notably, AP-1 and NFκB have been shown to directly correlate with CD44, by binding the CD44 promoter [16]. AP-1, a leucine zipper transcription factor consists of two families, JUN (c-JUN, JUNB and JUND) and Fos (c-Fos, FosB, Fra1 and Fra2). The Jun proteins can form homodimers with one another or heterodimers with the Fos proteins. Together these proteins bind to core sequences in the genome to regulate expression of a target gene. AP-1 is involved in a number of cellular processes similar to CD44 including differentiation, proliferation and apoptosis [17], [18]. Regulation by AP-1 is induced by growth factors, cytokines and oncoproteins, which are implicated in the proliferation and survival of cells. AP-1 activity in a cell, whether it be pro-apoptotic or pro-oncogenic, is determined by the composition of the homodimer or heterodimer formed as well as the tumor type and state of differentiation of the cell [18], [19].

NFκB, like AP-1, has been linked to the up-regulation of CD44, but no direct evidence has been shown. Increased HGF has been shown to enhance expression of CD44v6 through a complex of NFκB, c/EBPβ and EGR1 [20]. NFκB proteins have also been shown to be up-regulated in breast cancer stem cells (BCSCs), and their expressions have been correlated to increased expression of tumor stem cell markers, including CD44. Interestingly, the reduction of NFκB in a murine cell line Met-1 was able to reduce the number of CD44^+^/CD24^−/low^ cells [21].

Despite intense research on CD44, the mechanism by which the protein is up-regulated in cancer and BCSCs is not well understood. Gene regulatory elements, e.g., promoters and enhancers, recruit transcription factors and chromatin modifying proteins, and allow transcription of the target genes to occur [22]–[28]. Enhancers are required for both temporal and tissue/cell specific gene expression [22]–[28]. Therefore, it is an important task to identify and understand their role in gene expression of both normal and pathological conditions.

In this study, we report the identification of a novel *cis*-element of CD44 containing 717 bp (in human) and 715 bp (in mouse) of evolutionarily conserved noncoding DNA, located approximately 95 kb upstream of the CD44 transcription start site. We show that this *cis*-element has the ability to direct reporter gene expression in breast cancer cells in a cell type specific manner. These data suggest that this *cis*-element and its interacting transcription factors play an important role in regulating CD44 expression in breast cancer and BCSCs.

**Figure 1:**
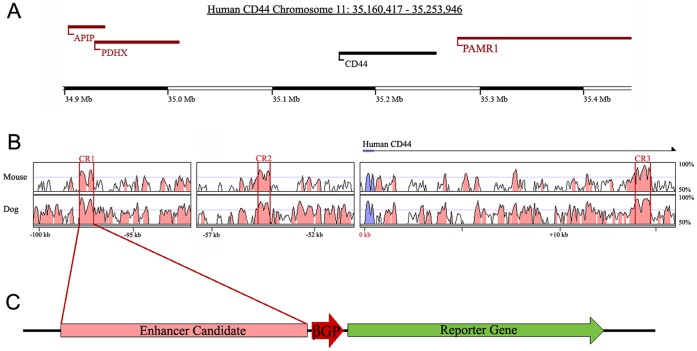
Prediction of *cis*-regulatory elements for CD44 expression using sequence alignment analysis. (**A**) A genomic map of human CD44 and surrounding genes located on chromosome 11p13. (**B**) Multiple sequence alignment of homologous CD44 sequences using human sequence as baseline. 14 evolutionarily conserved regions were identified and predicted as potential *cis*-regulatory elements for CD44 expression. Conserved regions 1–3 (CR1–3) have the highest levels of conservation. Blue regions represent CD44 coding sequence. Pink regions represent non-coding sequence. Peaks surrounded by red bars are highly conserved regions that have at least 70% conservation among species. (**C**) Plasmid reporter construct containing a conserved region of CD44, a minimal beta-globin-promoter (βGP), and green fluorescent protein (GFP).

## Materials and Methods

### Computational Prediction of CD44 *cis*-regulatory Elements

Multiple sequence alignment methods were used to identify evolutionarily conserved noncoding DNA sequences as putative gene regulatory elements. The sequences and annotations of analyzed genes along with their homologs from the various genomes were retrieved using noncoding sequence retrieval system, NCSRS [29]. These sequences were then aligned using multi-LAGAN [30] to identify elements with > 70% identity over a 100 bp span to ensure significance in sequence conservation. The percent identity and length of the CR were used to calculate a score for each conserved region (CR) (score = percent identity + (length/60)).

**Table 1:** Expression of key factors in 3 breast cancer cell lines.

	SUM159	MDA-MB-231	MCF7
Cell Type	Anaplastic Carcinoma	Epithelial-Adenocarcinoma	Epithelial- Adenocarcinoma
CD44	Very High	Very High	Low
CD24	Low	Negative/Low	High
Her2	Negative	Negative	Positive
PR	Negative	Negative	Positive
ER	Negative	ER (alpha−, beta+)	Positive
ALDH1	High	High	Low

**Figure 2:**
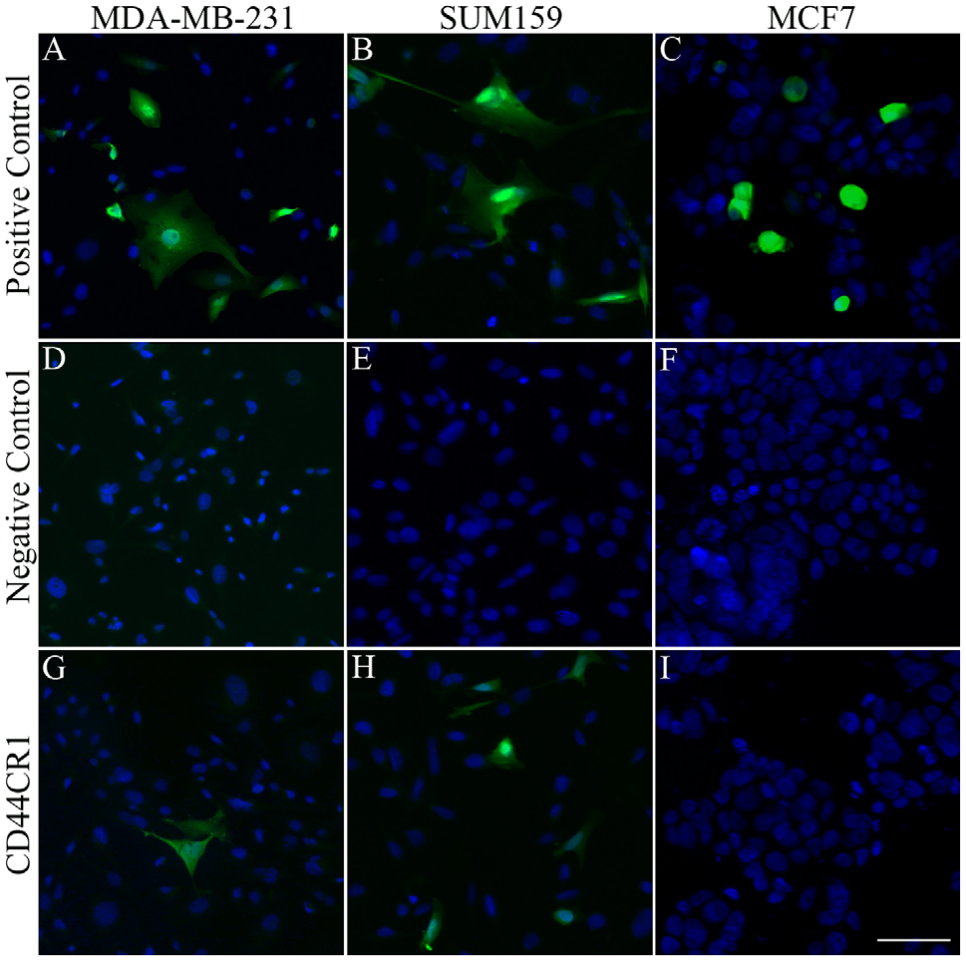
CR1 directs reporter GFP expression in breast cancer cell lines. Conserved region was tested for the ability to direct reporter gene expression by transfecting breast cancer cell lines with CD44CR1-βGP-GFP construct (CD44CR1-GFP). Nuclei were stained with Hoechst 33342. (**A–C**) GFP expression in all three cell lines resulted from transfection of a positive control construct (CAG-GFP). (**D–F**) No GFP expression was detected from transfection of a negative control construct with a conserved region from NeuroD1gene. GFP expression from CR1 can be seen in MDA-MB-231 and SUM159 cells (**G–H**). However, no expression is seen in MCF7 cells (**I**).

**Table 2:** Conserved transcription factor binding sites in CR1 between mouse and human.

Family	Matrix	From–to	Str.	Sequence
V$HAND	V$PARAXIS.01	95–115	(+)	cagaaACCAgatgtgttggtg
V$RP58	V$RP58.01	99–111	(−)	aacaCATCtggtt
V$RORA	V$REV-ERBA.02	115–137	(−)	tagaagctgaGTCAcaggatgac
V$AP-1R	V$NFE2.01	116–136	(−)	agaagCTGAgtcacaggatga
V$PBXC	V$PBX1_MEIS1.03	118–134	(−)	aagctgagTCACaggat
V$AP-1R	V$TCF11MAFG.01	118–138	(+)	atcctgTGACtcagcttctat
V$AP-1F	V$AP-1.01	122–132	(+)	tgtgACTCagc
V$AP-1F	V$AP-1.01	122–132	(−)	gctgAGTCaca
V$GATA	V$GATA.01	145–157	(+)	tgctGATAaataa
V$HOXC	V$PBX_HOXA9.01	145–161	(−)	ttctTTATttatcagca
V$PAX6	V$PAX6.02	159–177	(+)	gaagagtttCCAGgtatgc
V$BCL6	V$BCL6.02	161–177	(−)	gcataccTGGAaactct
V$STAT	V$STAT5.01	499–517	(+)	tttcTTCTtcgaagttccc
V$CAAT	V$NFY.03	176–190	(−)	taaaCCAAacatagc
V$NKXH	V$NKX31.01	203–217	(+)	gacagtAAGTatacc
V$SNAP	V$PSE.02	212–230	(+)	tatacCCTAaagttaccaa
V$HAML	V$AML3.01	241–255	(−)	ggttGTGGttcagag
V$EBOX	V$MYCMAX.02	259–271	(−)	tcaacaCATGtga
V$IRFF	V$IRF4.01	279–299	(+)	aaaagaaaaaGAAAaaagaaa
V$IRFF	V$IRF7.01	292–312	(+)	aaaaGAAAtgaaaattggaaa
V$OCT1	V$OCT1.06	296–310	(+)	gaaatgaaAATTgga
V$RBPF	V$RBPJK.02	508–522	(−)	cctaTGGGaacttcg
V$YBXF	V$YB1.01	518–530	(−)	cagatTGGCctat
V$CAAT	V$NFY.01	519–533	(+)	taggCCAAtctgtct
V$SP1F	V$GC.01	537–551	(−)	tgtggGGTGgggttg
V$CLOX	V$CDPCR3.01	585–607	(−)	gccctcagaaaaagatATTGctc
V$AP-1R	V$BACH2.01	609–629	(−)	aggcagTGAGtcagggtttac
V$AP-1R	V$NFE2.01	611–631	(+)	aaaccCTGActcactgcctcc
V$CREB	V$TAXCREB.02	611–631	(+)	aaacccTGACtcactgcctcc
V$CSEN	V$DREAM.01	612–622	(−)	gaGTCAgggtt
V$AP-1F	V$AP-1.01	615–625	(+)	cctgACTCact
V$AP-1F	V$AP-1.01	615–625	(−)	agtgAGTCagg
V$CARE	V$CARF.01	626–636	(+)	ggaagGAGGca
V$HAML	V$AML1.01	631–645	(−)	aactGTGGtaggaag
V$AIRE	V$AIRE.01	631–657	(−)	cagtgttttggaaactgTGGTaggaag
V$OCT1	V$POU2F3.01	671–695	(−)	tctATGCagatctcagt
V$OCT1	V$OCT3_4.02	671–695	(+)	gatctGCATagagacaa
V$FKHD	V$HNF3.01	703–719	(−)	tgtatgcAAACagctct
V$NFKB	V$NFKAPPAB.01	725–737	(+)	ctGGGAaatccct
V$NFKB	V$NFKAPPAB.01	726–738	(−)	aaGGGAtttccca
V$EVI1	V$EVI1.01	730–746	(−)	aagacAAGAagggattt

### Cell Culture

The breast cancer cell lines SUM159, MDA-MD-231 and MCF7, were describe previously [4]. SUM159 cells (Asterand Inc. Detroit, MI), MDA-MB-231 cells (ATCC), MCF7 cells (gift from Dr. Nanjoo Suh at Rutgers) were cultured according to the guidelines from the suppliers. All cell lines were maintained at 37°C in a humidified incubator with 5% CO_2_.

**Figure 3:**
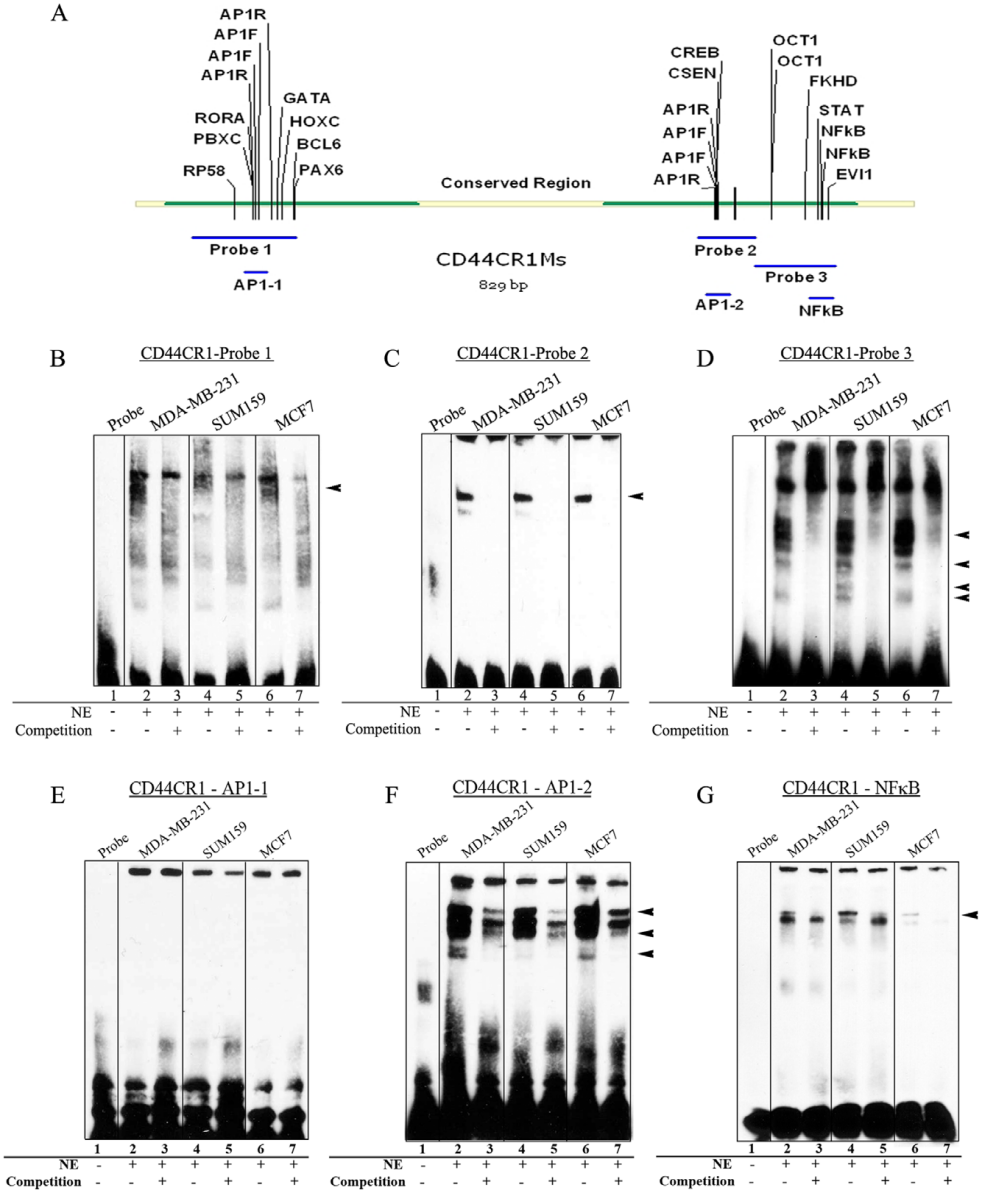
Specific protein factors bind with CR1. EMSAs were performed to determine the *in vitro* binding activities of nuclear protein factors with CD44CR1. (**A**) DNA probe design using conserved mouse sequence and TFBSs within each probe. Probe 1 identified binding (indicated by arrow head) in two cell lines (MDA-MB-231 and MCF7), but not observed in SUM159. (**B**) Probe 2 showed strong binding present in all three cell lines (arrowheads). (**C**) Probe 3 showed multiple shifted bands and was successfully competed away in all three cell lines using unlabeled probes. (**E**) Probe AP-1-1 showed no band shift in any of the three cell lines. (**F**) Probe AP-1-2 resulted in a band shift in all three cell lines. All band shifts were competed away with an unlabeled probe. Arrowheads indicate bands specific to MDA-MB-231 and MCF7. (**G**) Probe NFκB showed a band shift that was successfully competed away in all three cell lines.

**Figure 4:**
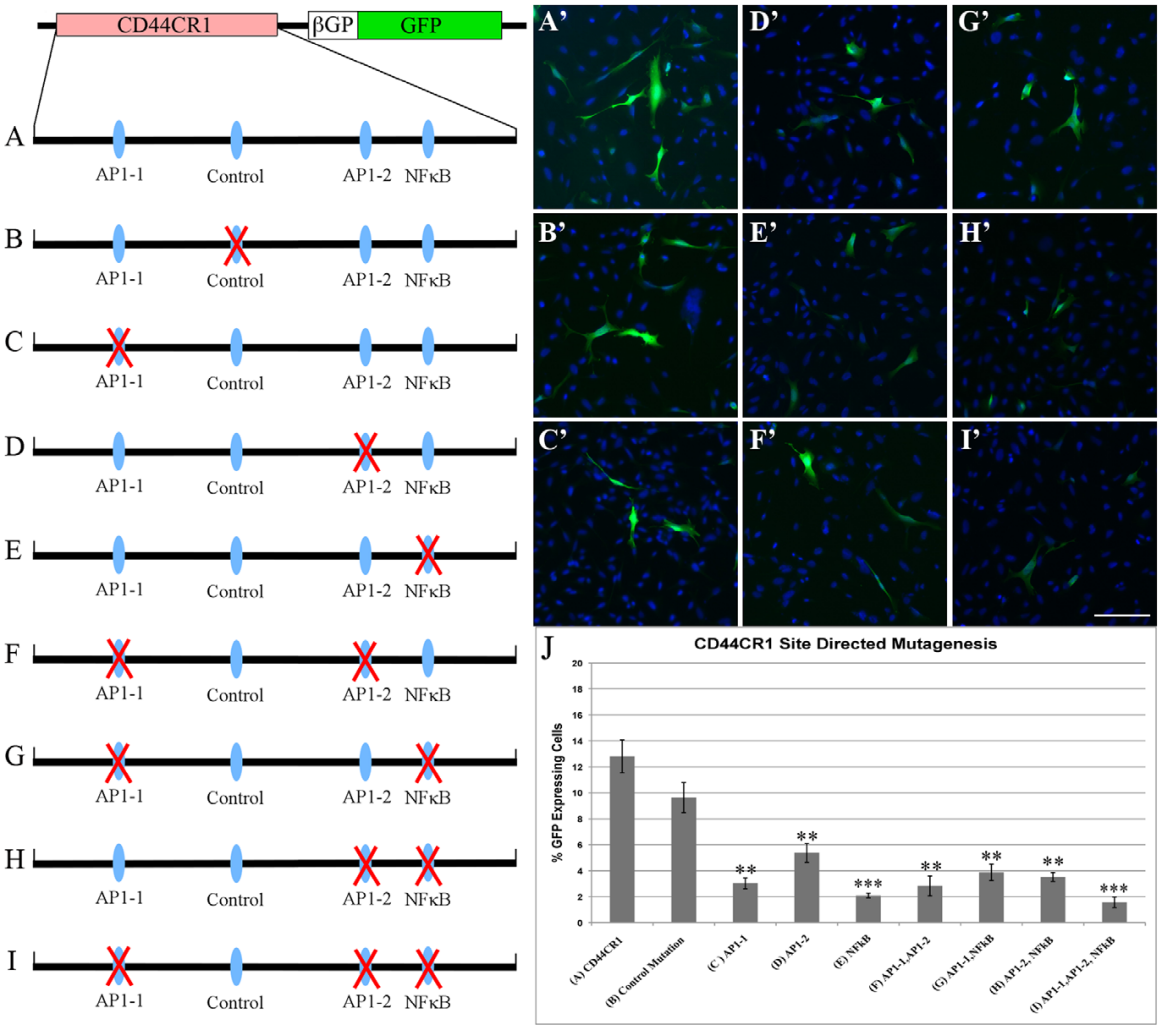
Mutation of AP-1 and NFκB binding sites in CR1 reduces reporter GFP expression. Assays using site directed mutagenesis of AP-1 and NFκB binding sites. (**A–I**) Schematic of each mutation of CR1 construct. Mutated sites are identified by a red X. (**A’–I’**) Transfection of each the constructs in SUM159 cells. (**J**) Quantification of the number of GFP-expressing cells/total number of cells counted. Control mutation at a non-conserved site (**B’**) showed no difference in GFP expression when compared to CR1 (**A’**). Single site mutations of AP-1-1, AP-1-2 and NFκB (**C’-E’**) showed a significant reduction of GFP expression compared to CR1. However, GFP expression was not eliminated entirely. Mutation of a combination of AP-1 and NFκB binding sites (**F’-H’**) did not reduce further GFP expression, however, the percentage of GFP expression was still significantly reduced compared to CR1. Mutation of all three TFBSs (**I’**) showed the greatest reduction of GFP expression. **p = < 0.0005 ***p = <1.0×10^−5^ (student’s t-test). Scale bar = 50 µM.

**Figure 5:**
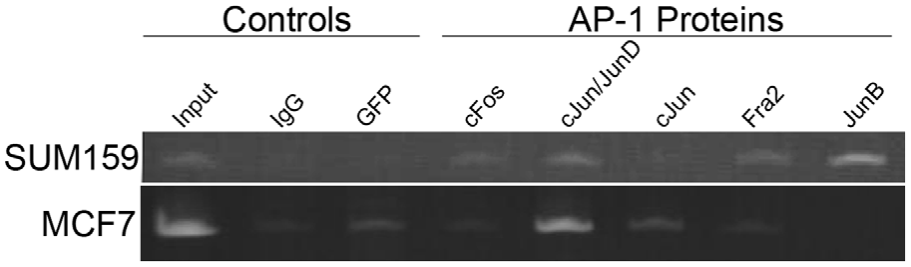
Differential AP-1 factor binding to CR1 in breast cancer cells. ChIP with AP-1 antibodies resulted in amplification of a region of CR1 with inverted repeat AP-1 binding sites. Rabbit IgG and anti-GFP antibody served as negative control. Representative results of at least two independent immunoprecipitation experiments and multiple independent PCR analyses are shown. Strong PCR amplification of CR1 region with JUNB binding was seen in SUM159 cells and with JUND binding in MCF7 cells.

**Figure 6:**
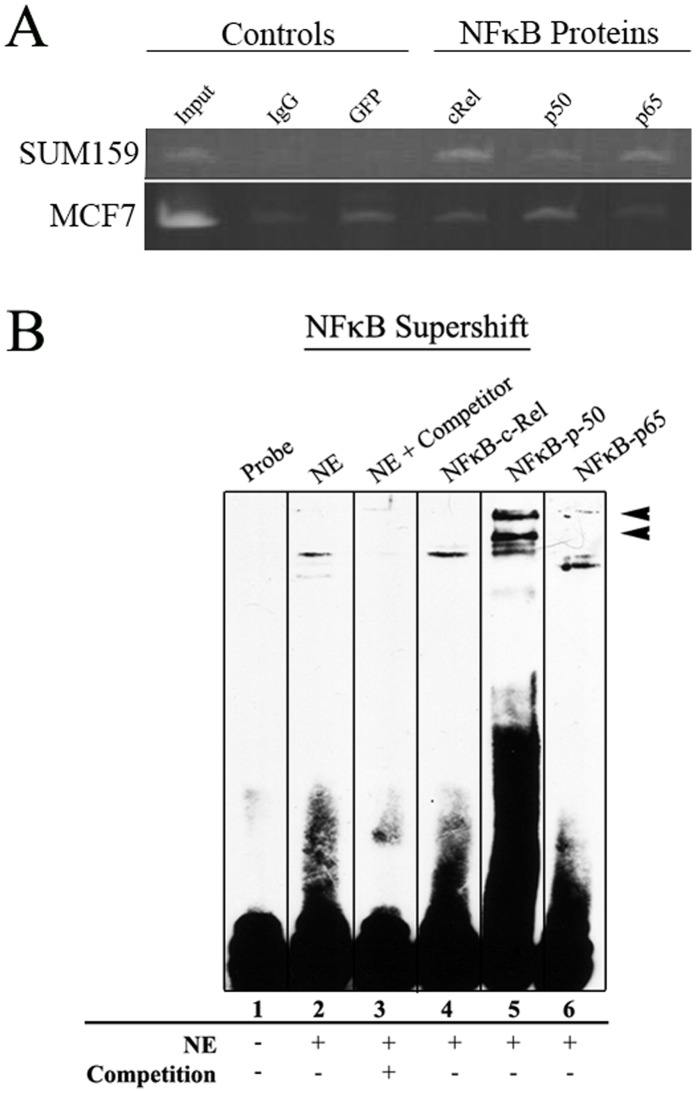
NFκB factors interact with CR1. ChIP assays were performed to identify CR1 interacting transcription factors. Rabbit IgG and anti-GFP antibody served as negative control. (**A**) Strong PCR amplification of CR1 region with NFκBp50 and p65 were seen in SUM159 samples. MCF7 samples showed bands with intensities equal to the negative control. (**B**) Supershift with NFκB antibodies was performed with SUM159 nuclear extract. Anti NFκB-p50 and p65 antibodies were able to supershift the band, but NFκB-cRel antibody resulted in no shift.

**Figure 7:**
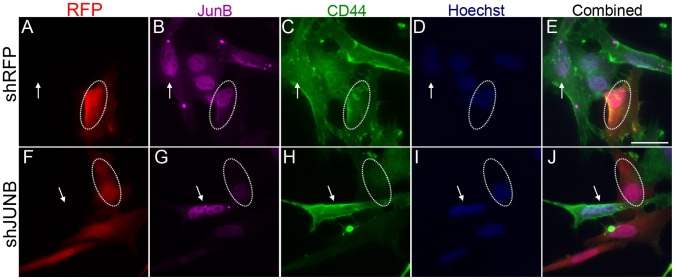
AP-1-JUNB knockdown decreases CD44 expression. Sum159 cells were transfected with control and JUNB shRNA constructs and then stained for JUNB and CD44 expression. Transfection with the control, scrambled DNA shRNA construct (**A–E**) showed no change in JUNB expression (**B**, circle) or CD44 expression (**C**, circle) when compared to un-transfected cells (arrows). Transfection with the JUNB shRNA construct (**F–J**) showed a reduction in JUNB expression (**G**, circle) and CD44 expression (**H**, circle) when compared to un-transfected cells (**F–G**, arrow).

**Figure 8:**
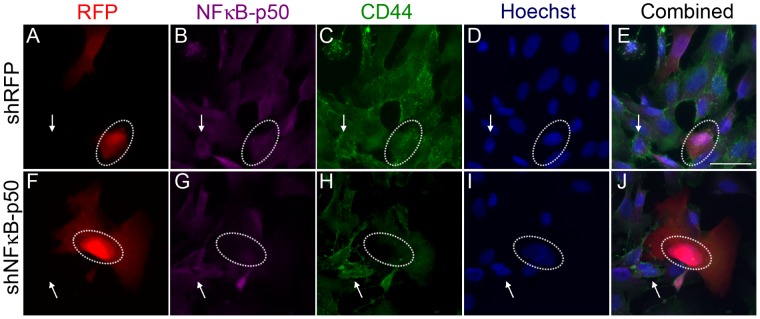
NFκB-p50 knockdown decreases CD44 expression. Sum159 cells were transfected with control and NFκB-p50 shRNA constructs and then stained for NFκB-p50 and CD44 expression. Transfection with the control, scrambled DNA shRNA construct (**A–E**) showed no change in NFκB-p50 expression (**B**, circle) or CD44 expression (**C**, circle) when compared to un-transfected cells (arrows). Transfection with the NFκB-p50 shRNA construct (**F–J**) showed a reduction in NFκB-p50 expression (**G**, circle) and CD44 expression (**H**, circle) when compared to un-transfected cells (**F–G**, arrow).

### Reporter Plasmids

Conserved regions were amplified by PCR from mouse genomic DNA (**Table S1**), subcloned into a GFP reporter plasmid with a basal beta-globin promoter (βGP-GFP) and verified by sequencing.

### Transfection

For transfections, cells were seeded onto poly-L-Lysine (PLL) treated coverslips in 24 well plates. Cells were transfected with Lipofectamine LTX (Invitrogen) as per manufacturer’s recommendations. Following a 24 hr incubation period, nuclei were stained with Hoechst33342 (Sigma). Cells were then fixed with 4% paraformaldehyde in PBS for 12 minutes at room temperature. Coverslips were adhered to slides with Fluoro-Gel (Electron Microscopy Sciences). GFP-expressing cells were visualized by a Zeiss AxioImager A1 fluorescence microscopy.

### qRT-PCR

RNA was isolated from cells using Tri Reagent (Ambion). cDNA was prepared by reverse transcription using the qScript cDNA SuperMix (Quanta), and used as a template for RT-PCR (PerfeCTa SYBR Green FastMix (Quanta)). RT-PCR reaction was run on a Roche LightCycler using primer sequences obtained from the Harvard Primer Bank (**Table S2**). Threshold cycles were normalized relative to GAPDH expression. Error bars represent the standard deviation of the mean.

### Data Quantification

In all experiments, percentages represent the averages calculated from at least three independent samples. All values are shown as a mean ± standard error of the mean. Error bars represent the standard error of the mean. In cases where results were tested for statistical significance, a student’s t-test was applied.

### Immunocytochemistry

For immunocytochemistry, cells were plated on PLL treated coverslips and incubated for 24 hours and then fixed to coverslips using 4% paraformaldehyde, blocked with 10% Donkey Serum (Jackson Immunology) and then incubated with the primary antibody for 2 hours at room temperature. The following antibodies were used [CD44 (Chemicon); CD24 (Santa Cruz); NFκB-c-Rel (Chemicon); NFκB-p50 (Upstate); NFκB-p65 (Abcam); JUNB (Santa Cruz); FosB (Santa Cruz)]. Following incubation with primary antibody, cells were incubated with a fluorescent secondary antibody (Jackson Immunology) for 30 minutes at room temperature. Nuclei were stained with Hoechst33342.

### Genomic DNA Sequencing

Genomic DNA was collected from the human cell lines using the Promega Genomic DNA kit as per manufacturer’s recommendations. Genomic DNA from each cell line was sequenced using primers specific for the conserved regions (**Table S1**). Genomic DNA was aligned using the online program ClustalW [31].

### Electrophoresis Mobility Shift Assay and Supershift

Single stranded DNA probes were designed from mouse CR1 and labeled with the 3’ Biotin End Labeling Kit (Thermo Scientific) as per manufacturer’s suggestions. Nuclear extracts were collected from each breast cancer cell line using NE-PER nuclear and cytoplasmic extraction reagents (Thermo Scientific). Binding reactions were performed and detected using the LightShift Chemiluminescent EMSA kit (Thermo Scientific) per manufacturer’s recommendations. DNA-protein complexes were run on 10% non-denaturing poly-acrylamide gels and transferred onto Biodyne Plus membrane (Pall). Membranes were cross-linked in a UV imager for 15 minutes. EMSA probe sequences are in **Table S3.** Supershift assays were performed in a similar fashion. Antibodies were added to select reactions 15 minutes prior to addition of labeled probes.

### Site Directed Mutagenesis

Site directed mutagenesis was performed as previously described [32] using primer sequences as listed in **Table S4**. Treated DNA was transformed into NEB5α cells (NEB) and plated onto LB-amp plates. Constructs were collected by Qiagen midi-prep and then sequenced to verify the resulting mutation. Mutated constructs were transfected into cells and tested for GFP expression.

### Chromatin Immunoprecipitation

Chromatin immunoprecipitation (ChIP) was performed as previously described [33], [34]. Sonication was performed using a Branson 450 Digital Sonicator. The chromatin extract was pre-cleared with protein A beads (NEB). NFκB-c-Rel (Chemicon); NFκB-p50 (Upstate); NFκB-p65 (Abcam); cJun(N) (Santa Cruz); cJun(D) (Santa Cruz); JUNB (Santa Cruz); FosB (Santa Cruz) antibodies were used to perform ChIP assay. Protein-DNA crosslinks were reversed with 30 µl 5 M NaCl and incubating samples at 65°C for 4 hours. Proteins were digested with 0.1 mM EDTA, 20 mM Tris-HCl and 2 µl Proteinase K solution (Active Motif) for 2 hours at 42°C. DNA was purified using phenol-chloroform extraction. PCR was performed to identify DNA:protein interactions. PCR primers used for ChIP assays are listed in **Table S5**.

### shRNA-based Gene Knockdown

Short hairpin RNA (shRNA) sequence (leading strand) used for AP1-JUNB knockdown were CCTTCTACCACGACGACTCATACACAGCT and CACGACTACAAACTCCTGAAACCGAGCCT. shRNA sequences for NFκB-p50 knockdown were GCAGCTCTTCTCAAAGCAGCAGGAGCAGA and GAGAACTTTGAGCCTCTCTATGACCTGGA (OriGene Technologies, Inc. , Rockville, MD). Control constructs were an empty vector and scrambled shRNA construct. Constructs were transfected into cell lines using Lipfectamine LTX (Life Technologies). Transfected cells were cultured for 72 hours before being fixed and stained as described above.

## Results

### Prediction of *cis*-regulatory Elements for CD44 Expression using Sequence Alignment Analysis

To understand the molecular mechanism of CD44 expression in breast cancer cells, highly conserved regions of non-coding DNA were computationally predicted as *cis*-regulators of CD44 expression. Multiple sequence alignment using the human CD44 genomic region as baseline revealed homologous regions in mouse, dog (**Fig. 1A**) and other mammalian species. A total of 14 conserved regions (CR) (>100 consecutive base pairs of sequence with >70% sequence identify) were identified. The three highest conserved regions (CR1–3, **Fig. 1B**) were chosen for further experimental verification, because many studies have shown that highly evolutionarily conserved noncoding DNA sequences have a high potential to regulate gene expression [35], [36]. CD44CR1 (CR1) contains 715 bp and located 95 kbp upstream of CD44 with 78% conservation. CR2 contains 611 bp with 76% conservation and is located 55 kbp upstream of CD44. CR3 contains 604 bp with 79% conservation and it is located in the first intron of the CD44 gene.

### Conserved Regions have the Ability to Direct Reporter GFP Expression in Breast Cancer Cells

To test the CRs for their ability to direct gene expression, the CRs were PCR amplified from mouse genomic DNA and subcloned into an expression vector containing a β-globin minimal promoter (βGP) and green fluorescent protein (GFP) as the reporter gene (**Fig. 1C**). Mouse DNA was used to validate that evolutionarily conserved elements can function in different species.

The ability of the conserved regions to direct gene expression was tested using three previously characterized human breast cancer cells, MDA-MB-231, SUM159, and MCF7, each with a different CD44/CD24 expression profile (**Table 1**) [4], [37]. Both MDA-MB-231 and SUM159 cells contain high levels of CD44 expression. In addition, SUM159 cells have been characterized with cancer stem cell like features including the ability to self-renew, reconstitute the parental cell line, survive chemotherapy, as well as form tumors with as few as 100 cells [4], [37]. Thus, these cells provide different lines of validation.

First, immunofluorescence staining was performed to verify CD44 and CD24 expression level. Consistent with the genome-wide expression profiling study [4], MDA-MB-231 and SUM159 cells showed very high CD44 staining and low CD24 staining, while MCF7 showed low CD44 and high CD24 staining (**Fig. S1A–C**).

Then, CD44 and CD24 expression level in the three cell lines was further quantified using quantitative PCR (qPCR). Results showed that MDA-MB-231 and SUM159 cells have the high CD44 and low CD24 expression, while MCF7 cells have the opposite expression profile, i.e., a higher CD24 and lower CD44 expression (**Fig. S1D**).

Next, each reporter construct containing one of the top three conserved regions of CD44 was individually tested by transfection into the three cell lines. Transfection of the positive control construct, CAG-GFP, resulted in reporter GFP expression (**Fig. 2A–C**) and demonstrated the ability of each of the cell lines to be transfected. As negative controls, a highly conserved region in Neurod1 locus with βGP and βGP alone (data not shown), resulted in no visible GFP expression (**Fig. 2D–F**)**,** indicating that not all highly conserved regions of genomic DNA nor βGP alone have the ability to direct gene expression. GFP expression was observed in MDA-MB-231 and SUM159 cell lines after transfection with CR1-GFP construct (**Fig. 2G–H**). More GFP-expressing cells were observed in SUM159 cells as compared to MDA-MB-231 cells, while no GFP-expressing cells were observed in MCF7 cells (**Fig. 2I**). Transfection of constructs containing CD44CR2 and CD44CR3 also resulted in GFP-expressing cells (data not shown, under further investigation).

### Analysis of Trans-acting Factor Binding Sites on the Conserved Regions of CD44

The ability of CR1 to direct different levels of reporter GFP expression among the three cell lines is most likely attributed to their interactions with *trans*-acting factors. Therefore, CR1 of both mouse and human were examined for *trans*-acting factor binding sites (TFBSs) and mutations in these sites. Genomic DNA of CR1 from each of the three cell lines was collected and sequenced to determine if mutations in the region that disrupt TFBSs. Sequencing results show only a 5 bp span that differed between the three human cell lines in CR1 (**Fig. S2**). This 5 bp difference found in the SUM159 cells is located in an unconserved region of CR1 and showed no disruption of key TFBSs. This indicates that the difference in GFP expression among these cells may not be associated with the DNA sequence. Thus, we speculate that the difference in GFP expression may be the result of *trans*-acting factor binding in the cell lines. CR1 sequences from mouse (**Table S6**) and human (**Table S7**) both contained over 150 putative TFBSs as predicted by MatInspector [38]. These TFBSs were examined further for conservation between mouse and human sequences (**Table 2**). Most of these conserved TFBSs involved in breast cancer (e.g., AP-1, NFκB, and STAT5), stem cells and embryonic development (e.g., OCT1, PAX6, GATA1), and therefore had the highest potential for regulating CD44 and for being involved in breast cancer. Our further analysis was thus focused on the activities of CR1 in regulating gene expression in breast cancer cells.

### Sequence Specific Trans-acting Factor Binding with CR1

Electrophoretic mobility shift assays (EMSAs) were performed to determine if differences in GFP expression resulted from differences in trans-acting factor binding in the cells. Double-stranded, biotin labeled oligonucleotides corresponding to subregions of CR1 were assayed for *trans*-acting factor binding using nuclear extract from each of the three cell lines (**Fig. 3A**). The shifted bands for three of the large probes spanning the length of the conserved regions in all three cell types (**Fig. 3B–D**) indicating protein-DNA binding activity. Probe 1 shows strong bands shifted with nuclear extracts from MDA-MB-231 and MCF7 cells only (**Fig. 3B**), while probe 2 has a band shifted that is equally strong with all three cell lines (**Fig. 3C**). Probe 3 shows a number of bands that can be competed away with an unlabeled probe (**Fig. 3D**). Although the bands in probe 3 are similar in all three cell lines, there was a band with SUM159 cells that is not present in the other two cell lines.

Smaller probes were then used to narrow down regions of binding and to identify specific TFBSs. A probe designed to mimic the first AP-1 site (AP-1-1) showed no band shift (**Fig. 3E**), while the probe for the second AP-1 site (AP-1-2) showed a number of band shifts (**Fig. 3F**). Although these bands were not completely competed away, there was a significant reduction in band intensity with the addition of the competition probe. A probe for the region of NFκB binding also revealed band shifts. The intensity of the band differed among cell lines, with SUM159 showing the strongest shift (**Fig. 3G**).

### Mutation of AP-1 and NFκB Binding Sites Results in a Loss of CR1 Expression

EMSA identified regions of CR1 that were able to bind nuclear factors in each of the three cell lines. However, these *in vitro* assays are not sufficient to determine if these factors have the ability to direct gene expression. To determine if the specific TFBSs are involved in the regulation of reporter GFP expression, site directed mutagenesis (SDM) was performed. The core binding sites for the two AP-1 TFBSs and NFκB binding site were deleted from the CR1 reporter construct using SDM. Mutant constructs were transfected into each of the cell lines. Wild-type CR1 and a random mutation were used as control transfections. Results show that the control transfections no significant difference in the percentage of GFP-expressing cells (**Fig. 4A–B**), whereas single site mutations at each AP-1 site and NFκB binding site (**Fig. 4C–E**) resulted in statistically significant decrease in the percentage of GFP-expressing cells in SUM159 cell line when compared to un-mutated CR1 and the control mutation (**Fig. 4A–B**).

Since GFP expression was not completely abolished with the deletion of a single TFBS in SUM159, we mutated a combination of TFBSs (**Fig. 4F–H**). Results of transfections with combinatorial mutations again showed a statistically significant decrease in the percentage of GFP-expressing cells (**Fig. 4F–H**). However, the percentage of GFP-expressing cells with two mutation constructs did not change significantly as compared with single-mutation constructs. To determine whether all three sites are needed for CR1 to direct GFP expression, the three binding sites were mutated (**Fig. 4I**). The transfection of this construct resulted in the highest decrease in the percentage of GFP-expressing cells. Interestingly, transfection of the mutant constructs into MDA-MB-231 resulted in no GFP-expressing cells (**Fig. S3**) suggesting regulation of CD44 in MDA-MB-231 differs from SUM159 cells.

*Trans-acting* factor binding assays identify components of AP-1 and NFκB binding to CR1 in SUM159 cells.

To determine whether the difference in reporter GFP expression among the three breast cancer cells is due to the *trans*-acting factors binding with CR1, chromatin immunoprecipitation (ChIP) assays were performed using antibodies against individual components of AP-1 and NFκB. ChIP results show that in SUM159 cells JUNB bound strongly with CR1, while in MCF7 cells only JUND bound to CR1 (**Fig. 5**). When ChIP assays were performed with antibodies against NFκB components (e.g., c-Rel, p50 and p65), SUM159 revealed weak binding with all three NFκB antibodies (**Fig. 6A**). However, MCF7 showed no significant binding when compared to background. These results are supported by an EMSA supershift assay performed to verify specific proteins binding using antibodies against NFκB proteins c-Rel, p50 and p65 (**Fig. 6B**). The antibody against NFκB-p50 was able to provide a significant shift in the labeled probe. NFκB-p65 showed a weaker shift similar to NFκB-p50 as well as a band that was downshifted. Together these results support the notion that the different cell lines have different means by which they regulate CD44.

### JUNB and NFκB-p50 Knockdown Represses CD44 Expression

To determine the effects of AP-1-JUNB and NFκB-p50 on CD44 expression, we performed shRNA gene knockdown experiments in SUM159 cells. Control transfections, with scrambled control shRNA (**Fig. 7A–E**) or an empty vector (**Fig. S3A–E**), showed no change in JUNB or CD44 expression in transfected cells. Transfection of shJUNB constructs resulted in a decreased JUNB expression as shown by immunocytochemistry (**Fig. 7F–J** and **Fig. S3F–J**). Cells transfected with the shJUNB construct also showed a decrease in CD44 expression as compared to untransfected cells (**Fig. 7**). Similar results were seen with knockdown of NFκB-p50. Control shRNA transfection with a scrambled shRNA (**Fig. 8A–E**) or empty shRNA construct (**Fig. S4A–E**) showed no change in NFκB-p50 or CD44 expression. Knockdown of NFκB-p50 (**Fig. 8F–J** and **Fig. S4F–J**) did result in a decrease in CD44 expression compared to untransfected cells. These results support the notion that JUNB and NFκBp50 interact with CR1 and regulate CD44 expression.

## Discussion

In breast cancer, the up-regulation of CD44, a cell surface glycoprotein involved in cell-cell and cell-extracellular matrix adhesion, migration, differentiation and survival, is associated with cancer stem cells [39], [40]. However, the mechanism for this gene up-regulation is not well understood. In this study, we identified the novel *cis*-element CR1, with the ability to direct reporter gene expression in a cell specific manner (**Fig. 2**), and the *trans*-acting factors AP-1 and NFκB as key factors involved in the regulation of CR1 (**Fig. 3**).

Genomic sequencing of CR1 from breast cancer cell lines did not reveal any major mutations that cause changes in key TFBSs (**Fig. S2**), which suggests that variations in reporter gene expression among these cells may be attributed to the difference in *trans*-acting factor binding to CR1.

Consistent with the notion that there was a difference in *trans*-acting factor(s) binding to CR1, mutations of TFBSs for AP-1and NFκB resulted in a significant reduction in GFP expression in two breast cancer cell lines (**Fig. 4**). Deletion of each site individually was able to completely eliminate reporter gene expression in MDA-MB-231 (**Fig. S4**). However, deletion of all three sites TFBS, individually and sequentially in SUM159 cells did not completely eliminate reporter gene expression (**Fig. 4**). These results indicate that factors AP-1 and NFκB are important *trans*-regulators of gene expression in breast cancer; and AP-1 and NFκB function in a cell type specific manner via various binding patterns to CR1 in different breast cancer cell lines. The inability to completely eliminate CR1 expression implies other TFs and/or co-factors may be involved in regulating CD44 expression in breast cancer stem-like SUM 159 cells.

Our ChIP results showed that binding of AP-1 with CR1 in SUM159 and MCF7 cells, however, the two cells showed a different pattern of TF binding to CR1, i.e., JUNB in SUM159 and JUND in MCF7 (**Fig. 5**). ChIP results also showed that NFκB factors cRel, p50 and p65 bind to CR1 in SUM159 cells but not MCF7. This result was confirmed with an EMSA supershift with SUM159 nuclear extract, showing shifts with both NFκB-p50 and p65 (**Fig. 6**).

The observation that knockdown of AP-1-JUNB and NFκB-p50 reduced the expression of CD44 suggest the role of JUNB and p50 in regulating CD44 expression via their interaction with CR1. The fact that a complete loss of CD44 expression was not seen may be attributed to 1) reduced JUNB and p50 expression as opposed to a complete knockdown; 2) other factors interact with JUNB and/or p50 in the regulation of CD44 expression; and 3) other regulatory regions allowing basal expression of CD44.

Studies have shown that deletion of CD44 can lead to a reduction in recurrence of cancers [5] and metastasis [41]. By targeting the factors that result in the overexpression of CD44, we may be able to better treat breast cancer and metastatic tumors.

Previous studies have shown that AP-1 regulates CD44 expression [18], [42]–[44]. AP-1 has an increased activity in small cell and non-small cell lung carcinomas, which lead to an increase in CD44 expression. In addition, a TRE binding element with Fra-1 in the promoter of CD44 has been identified [45], [46]. These studies have established that AP-1 regulates CD44 expression via its interaction with CD44 promoter. In this study, our findings suggest that the *cis*-element CR1 functions via common factor AP-1 and/or NFκB and interact with the promoter to regulate CD44 expression, which provides new insight into regulatory mechanisms on complex CD44 expression.

Together, our findings suggest that CR1 has the potential to regulate CD44 expression in breast cancer and BCSCs via its interaction with AP-1 and NFκB factors. Further studies will focus on how CR1 interacts with the promoter to regulate CD44 expression. CD44 is known to have a complex expression patterns with ubiquitous expression and variant forms, and has been implicated in the aggressiveness and metastasis of a number of cancer types [9], [11], [37], [47]. Therefore, the regulation of such a molecule could be equally complex. A full understanding of complex regulation of CD44 expression requires the investigation of the other *cis*- and *trans*-regulators of CD44.

## Supporting Information

Figure S1 **CD44 and CD24 expression in breast cancer cell lines as detected by immunocytochemistry.** Human cell lines MDA-MB-231 (a–a’’’), SUM159 (b–b’’’), and MCF7 (c–c’’’) were fixed and stained for CD44 (F10442, Millipore) and CD24 (91, Millipore). Nuclei were stained with Hoechst33342. D. Real-time PCR analysis of CD44 and CD24 mRNA levels in breast cancer cell lines. GAPDH served as endogenous control. Immunohistochemistry and Real-time PCR showed high CD44 and low CD24 expression in MDA-MB231 and SUM159 cell lines. MCF7 cells showed low CD44 and high CD24 expression. Scale bar = 100 µm.
(TIF)

Figure S2 **Genomic sequence alignment of conserved regions reveals no mutations in TFBSs.** Genomic DNA was obtained from the cell lines MDA-MB-231, SUM159 and MCF7. Genomic DNA was sequenced at CD44CR1 conserved region and aligned using ClustalW. Alignment of CD44CR1 sequences identified a 5 bp deletion located in SUM159 genomic DNA. However, these mutations do not change TFBSs.
(TIF)

Figure S3 **JUNB knockdown decreases CD44 expression.** Sum159 cells were transfected with control and JUNB shRNA constructs and then stained for JUNB and CD44 expression. Transfection with the control, empty vector shRNA construct (A–E) showed no change in JUNB expression (B, circle) or CD44 expression (C, circle) when compared to un-transfected cells (arrows). Transfection with the JUNB shRNA construct (F–J) showed a reduction in JUNB expression (G, circle) and CD44 expression (H, circle) when compared to un-transfected cells (F–G, arrow).
(TIF)

Figure S4 **NFκBp50 knockdown decreases CD44 expression.** Sum159 cells were transfected with control and NFκB-p50 shRNA constructs and then stained for NFκB-p50 and CD44 expression. Transfection with the control, empty vector shRNA construct (A–E) showed no change in NFκB-p50 expression (B, circle) or CD44 expression (C, circle) when compared to un-transfected cells (arrows). Transfection with the NFκB-p50 shRNA construct (F–J) showed a reduction in NFκB-p50 expression (G, circle) and CD44 expression (H, circle) when compared to un-transfected cells (F–G, arrow).
(TIF)

Table S1 **PCR Primers for the amplification of the three conserved regions.**
(DOC)

Table S2 **qPCR primer sequences obtained from Harvard Primer Bank.**
(DOC)

Table S3 **Probe design for EMSA.**
(DOC)

Table S4 **Primers used for site directed mutagenesis.**
(DOC)

Table S5 **Primers used for ChIP assays.**
(DOC)

Table S6 **Predicted transcription factor binding sites from mouse CD44CR1.**
(DOC)

Table S7 **Predicted transcription factor binding sites from human CD44CR1.**
(DOC)
