# Supplementary figures and images for: Cell Specific CD44 Expression in Breast Cancer Requires the Interaction of AP-1 and NFκB with a Novel cis-Element
Source: PLoS One. 2012 Nov 30;7(11):e50867. doi: 10.1371/journal.pone.0050867 (PMC3511339; doi:10.1371/journal.pone.0050867)

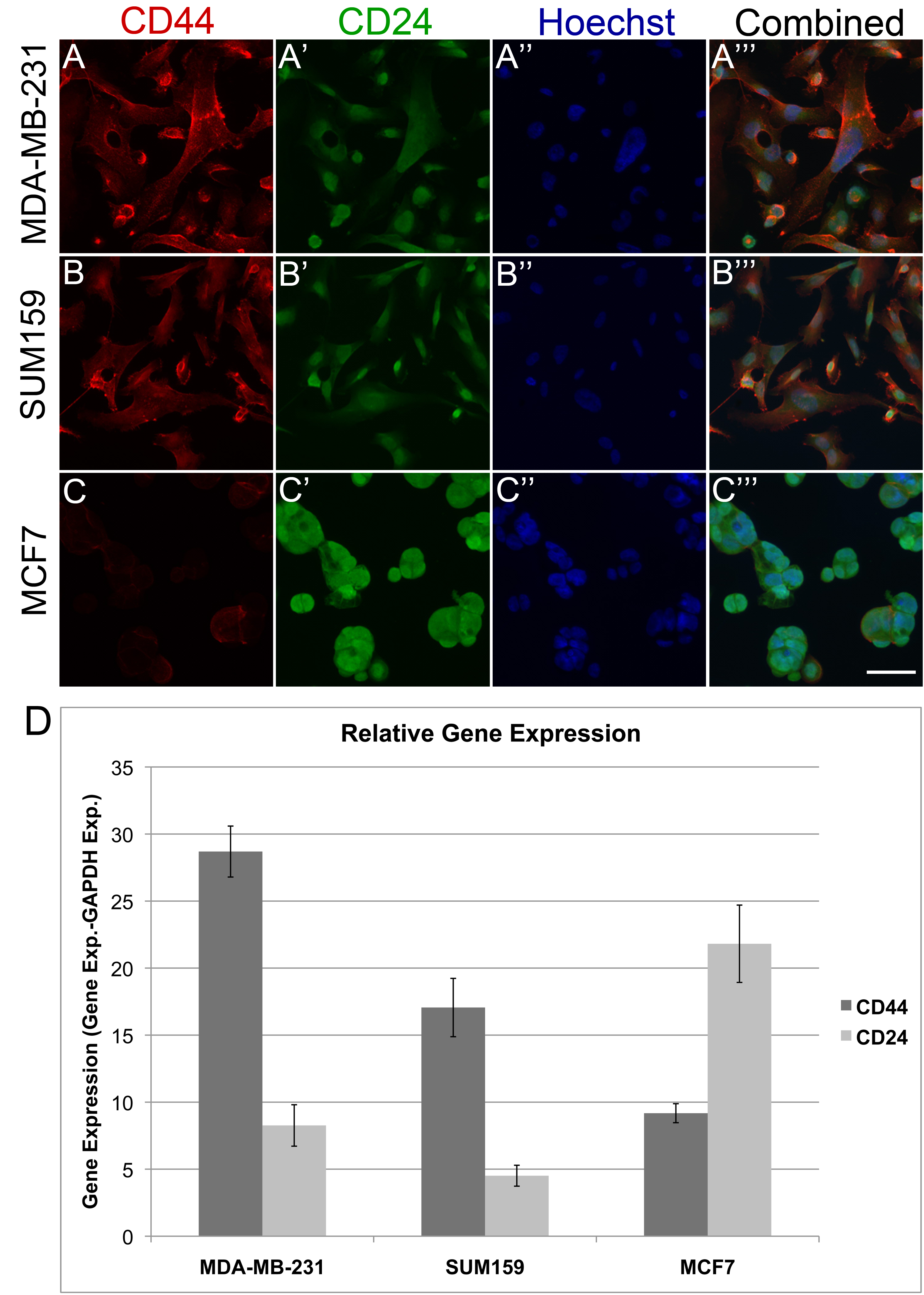

Supplement: Figure S1 — CD44 and CD24 expression in breast cancer cell lines as detected by immunocytochemistry. Human cell lines MDA-MB-231 (a–a’’’), SUM159 (b–b’’’), and MCF7 (c–c’’’) were fixed and stained for CD44 (F10442, Millipore) and CD24 (91, Millipore). Nuclei were stained with Hoechst33342. D. Real-time PCR analysis of CD44 and CD24 mRNA levels in breast cancer cell lines. GAPDH served as endogenous control. Immunohistochemistry and Real-time PCR showed high CD44 and low CD24 expression in MDA-MB231 and SUM159 cell lines. MCF7 cells showed low CD44 and high CD24 expression. Scale bar = 100 µm. (TIF) [file pone.0050867.s001.tif]

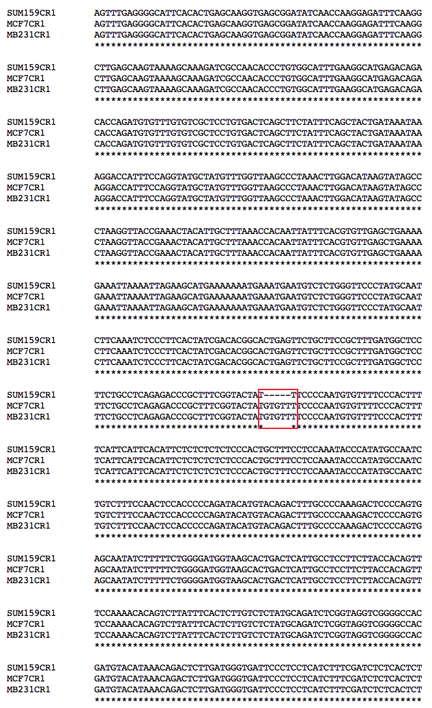

Supplement: Figure S2 — Genomic sequence alignment of conserved regions reveals no mutations in TFBSs. Genomic DNA was obtained from the cell lines MDA-MB-231, SUM159 and MCF7. Genomic DNA was sequenced at CD44CR1 conserved region and aligned using ClustalW. Alignment of CD44CR1 sequences identified a 5 bp deletion located in SUM159 genomic DNA. However, these mutations do not change TFBSs. (TIF) [file pone.0050867.s002.tif]

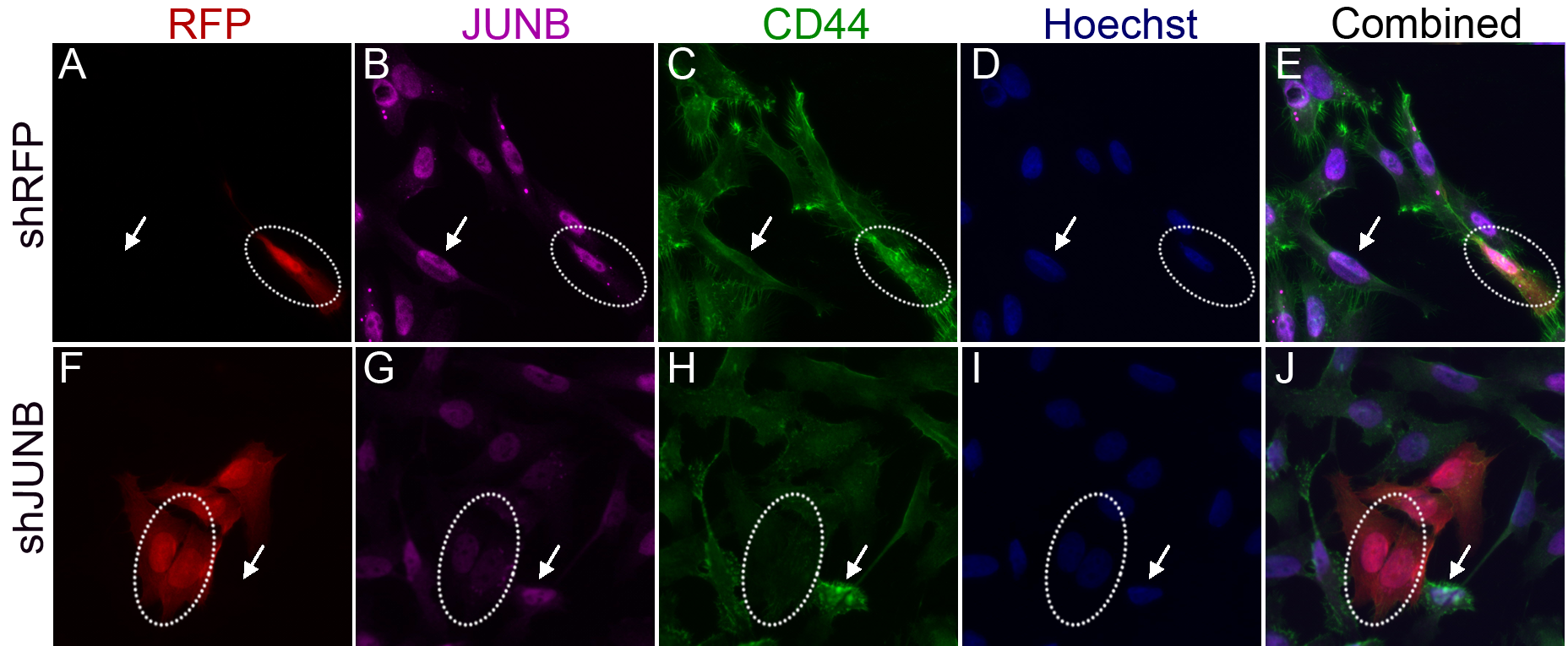

Supplement: Figure S3 — JUNB knockdown decreases CD44 expression. Sum159 cells were transfected with control and JUNB shRNA constructs and then stained for JUNB and CD44 expression. Transfection with the control, empty vector shRNA construct (A–E) showed no change in JUNB expression (B, circle) or CD44 expression (C, circle) when compared to un-transfected cells (arrows). Transfection with the JUNB shRNA construct (F–J) showed a reduction in JUNB expression (G, circle) and CD44 expression (H, circle) when compared to un-transfected cells (F–G, arrow). (TIF) [file pone.0050867.s003.tif]

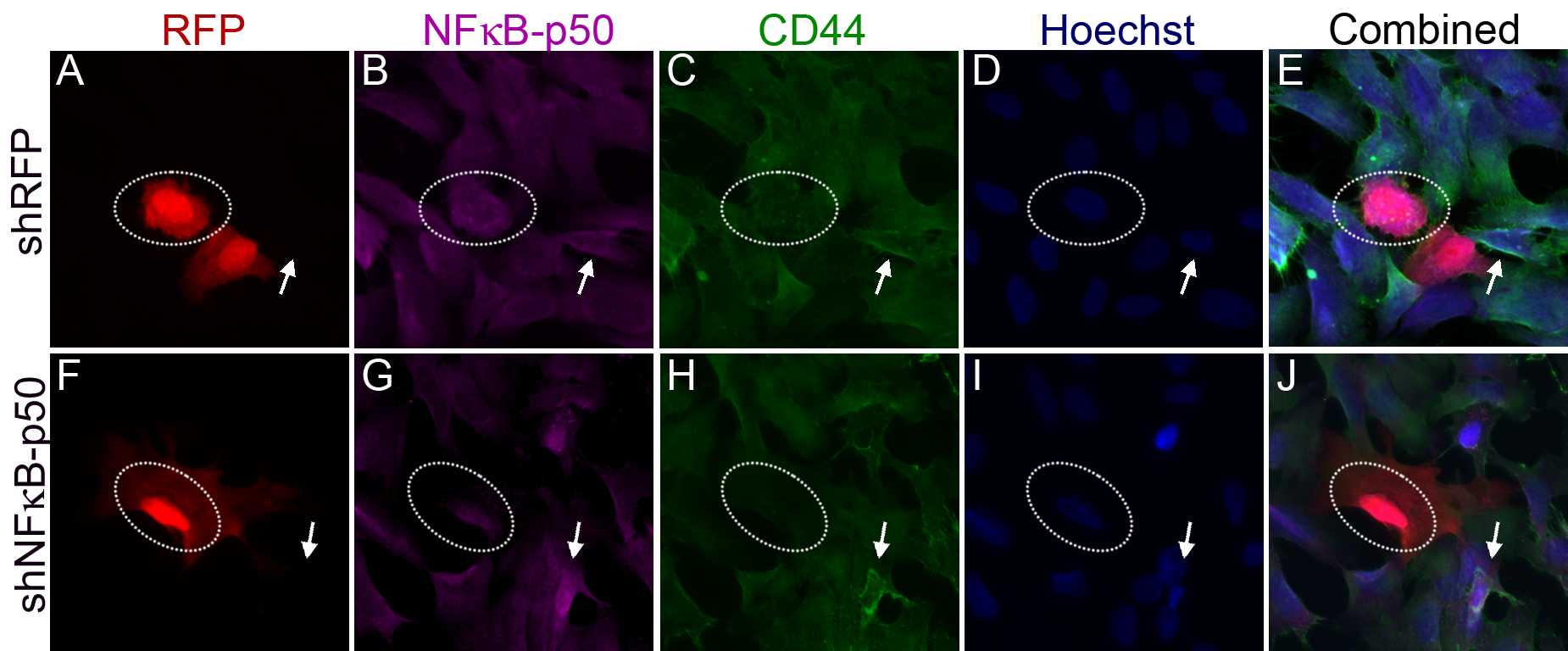

Supplement: Figure S4 — NFκBp50 knockdown decreases CD44 expression. Sum159 cells were transfected with control and NFκB-p50 shRNA constructs and then stained for NFκB-p50 and CD44 expression. Transfection with the control, empty vector shRNA construct (A–E) showed no change in NFκB-p50 expression (B, circle) or CD44 expression (C, circle) when compared to un-transfected cells (arrows). Transfection with the NFκB-p50 shRNA construct (F–J) showed a reduction in NFκB-p50 expression (G, circle) and CD44 expression (H, circle) when compared to un-transfected cells (F–G, arrow). (TIF) [file pone.0050867.s004.tif]
