# Supplementary material for: Cell Specific CD44 Expression in Breast Cancer Requires the Interaction of AP-1 and NFκB with a Novel cis-Element
Source: PLoS One. 2012 Nov 30;7(11):e50867. doi: 10.1371/journal.pone.0050867 (PMC3511339; doi:10.1371/journal.pone.0050867)
Supplement: Table S1 — PCR Primers for the amplification of the three conserved regions. (DOC) [file pone.0050867.s005.doc]

**Table S1. PCR Primers for the amplification of the three conserved regions.**

| **Conserved Region** | **PCR product length (bp)** | **Primer** | **Mouse Sequence** | **Human Sequence** |
| --- | --- | --- | --- | --- |
| **CD44CR1** | 829 | Forward | GGGCAGGATGAGTGGTTATTGAGA | GGTGAAATGCCCTATAGCTCAACTCTG |
| (715 bp) |  | Reverse | GGGTGGAATACAACCACACTGCAT | GTGCTTATTTCACATTGCATTCCTGC |
| **CD44CR2** | 735 | Forward | CACTGTTTGAAATGGGTGGCGATG | TGCTGCAATATAGACTTTCTGACC |
| (611 bp) |  | Reverse | GCATGAAACCACAGAGCCTACAGA | GACTGTCGTGTTTGTTCTCACTC |
| **CD44CR3** | 732 | Forward | TCCTACCTGTCTCCAGTGTTGTGA | TGGGCCCAGCTCAGTTTATACCTT |
| (604 bp) |  | Reverse | AACAACATTCCACAGACTGGCTCG | GGTCCCTTCTTCCCATCAGTTTCT |
