# Supplementary material for: Cell Specific CD44 Expression in Breast Cancer Requires the Interaction of AP-1 and NFκB with a Novel cis-Element
Source: PLoS One. 2012 Nov 30;7(11):e50867. doi: 10.1371/journal.pone.0050867 (PMC3511339; doi:10.1371/journal.pone.0050867)
Supplement: Table S2 — qPCR primer sequences obtained from Harvard Primer Bank. (DOC) [file pone.0050867.s006.doc]

**Table S2. qPCR primer sequences obtained from Harvard Primer Bank.**

| **Name** | **Primer** | **Sequence** |
| --- | --- | --- |
| **CD44** | Forward | TGCCGCTTTGCAGGTGTATT |
|  | Reverse | CCGATGCTCAGAGCTTTCTCC |
| **CD24** | Forward | CTCCTACCCACGCAGATTTATTC |
|  | Reverse | AGAGTGAGACCACGAAGAGAC |
| **GAPDH** | Forward | CATGAGAAGTATGACAACAGCCT |
|  | Reverse | AGTCCTTCCACGATACCAAAGT |
