# Supplementary material for: Cell Specific CD44 Expression in Breast Cancer Requires the Interaction of AP-1 and NFκB with a Novel cis-Element
Source: PLoS One. 2012 Nov 30;7(11):e50867. doi: 10.1371/journal.pone.0050867 (PMC3511339; doi:10.1371/journal.pone.0050867)
Supplement: Table S3 — Probe design for EMSA. (DOC) [file pone.0050867.s007.doc]

**Table S3. Probe design for EMSA**

| **EMSA Probes** | **Forward Sequence** |
| --- | --- |
| **CD44CR-60-170** | gattgccaacacccaggaaataaggaagaatgagacagaaaccagatgtgttggtgtcatcctgtgactcagcttctattctggttgctgataaataaagaagagtttcca |
| **CD44CR1-600-660** | ctgagggcagtaaaccctgactcactgcctccttcctaccacagtttccaaaacactgcta |
| **CD44CR1-660-745** | attgcgcccttgtctctatgcagatctcagtcagtctgggccaccatgtatgcaaacagctctttctgggaaatcccttcttgtct |
| **CD44CR1-600-745** | ctgagggcagtaaaccctgactcactgcctccttcctaccacagtttccaaaacactgctattgcgcccttgtctctatgcagatctcagtcagtctgggccaccatgtatgcaaacagctctttctgggaaatcccttct |
| **CD44CR1-450-495** | ccagtgggtttccccacctttccttcactcacatctctctctcccc |
| **CD44CR1-490-525** | ctccccgactttcttcttcgaagttcccataggcca |
| **CD44CR1-550-590** | catgcatgtacagacttcgtccgaagcctccctgtgagca |
| **CD44CR1-AP-1-1** | tcatcctgtgactcagcttctatt |
| **CD44CR1-AP-1-2** | gtaaaccctgactcactgcctcct |
| **CD44CR1-NFκB** | ctctttctgggaaatcccttcttgt |
| **CD44CR1-ETS-1** | aacacccaggaaataaggaagaatgagac |
| **CD44CR1-ETS-2** | gttggtgtcatcctgtgactc |
