# Supplementary material for: Cell Specific CD44 Expression in Breast Cancer Requires the Interaction of AP-1 and NFκB with a Novel cis-Element
Source: PLoS One. 2012 Nov 30;7(11):e50867. doi: 10.1371/journal.pone.0050867 (PMC3511339; doi:10.1371/journal.pone.0050867)
Supplement: Table S4 — Primers used for site directed mutagenesis. (DOC) [file pone.0050867.s008.doc]

**Table S4. Primers used for site directed mutagenesis.**

| **Name** | **Primer** | **Sequence** |
| --- | --- | --- |
| **CD44CR1ΔAP-1-1** | Forward | GGTGTCATCCTGTGAGCTTCTATTCTGG |
|  | Reverse | CCAGAATAGAAGCTCACAGGATGACACC |
| **CD44CR1ΔAP-1-2** | Forward | GGCAGTAAACCCTCACTGCCTCCTTCCTACC |
|  | Reverse | GGTAGGAAGGAGGCAGTGAGGGTTTACTGCC |
| **CD44CR1ΔNFκB** | Forward | CAAACAGCTCTTTCTAATCCCTTCTTGTC |
|  | Reverse | GACAAGAAGGGATTAGAAAGAGCTGTTTG |
| **SDM Control Deletion** | Forward | CCATGGGCTTTCCACATGGTAAATGTCCCTTTGC |
|  | Reverse | GCAAAGGGACATTTACCATGTGGAAAGCCCATG |
