# Supplementary material for: Cell Specific CD44 Expression in Breast Cancer Requires the Interaction of AP-1 and NFκB with a Novel cis-Element
Source: PLoS One. 2012 Nov 30;7(11):e50867. doi: 10.1371/journal.pone.0050867 (PMC3511339; doi:10.1371/journal.pone.0050867)
Supplement: Table S5 — Primers used for ChIP assays. (DOC) [file pone.0050867.s009.doc]

**Table S5: Primers used for ChIP assays**

| **ChIP Probes CD44CR-AP-1-1 – 373 bp** | |
| --- | --- |
| CD44CR1 – AP-1-1 Forward | AGGTGAGCGGATATCAACCAAGGA |
| CD44CR1 – AP-1-1 Reverse | AGAACTCAGTGCCGTGTCGATAGT |
| **ChIP Probes CD44CR1-NFκB – 362bp** | |
| CD44CR1 – NFκB Forward | CCAGGTATGCTATGTTTGGTTAAGCCC |
| CD44CR1 –NFκB Forward | GTGGAGTTGGAAAGACAGATTGGC |
| **ChIP Probes CD44CR1-AP-1-2 – 400bp** | |
| CD44CR1 – AP-1-1 Forward | TCTCTCCCACTGCTTTCCTCCAAA |
| CD44CR1 – AP-1-1 Reverse | GTGCTTATTTCACATTGCATTCCTGC |
