# Supplementary material for: Cell Specific CD44 Expression in Breast Cancer Requires the Interaction of AP-1 and NFκB with a Novel cis-Element
Source: PLoS One. 2012 Nov 30;7(11):e50867. doi: 10.1371/journal.pone.0050867 (PMC3511339; doi:10.1371/journal.pone.0050867)
Supplement: Table S6 — Predicted transcription factor binding sites from mouse CD44CR1. (DOC) [file pone.0050867.s010.doc]

**Table S6. Predicted transcription factor binding sites from mouse CD44CR1.**

| **Matrix Family** | **Detailed Family Information** | **Matrix** | **Matrix sim.** | **Sequence** |
| --- | --- | --- | --- | --- |
| V$EVI1 | EVI1-myleoid transforming protein | V$EVI1.01 | 0.733 | aagacAAGAagggattt |
| V$NFKB | Nuclear factor kappa B/c-rel | V$NFKAPPAB.01 | 0.957 | ctGGGAaatcccttc |
| V$NFKB | Nuclear factor kappa B/c-rel | V$NFKAPPAB.01 | 0.994 | aaGGGAtttcccaga |
| V$IKRS | Ikaros zinc finger family | V$IK1.01 | 0.941 | ttctGGGAaatcc |
| V$RBPF | RBPJ - kappa | V$RBPJK.02 | 0.954 | tttcTGGGaaatc |
| V$STAT | Signal transducer and activator of transcription | V$STAT.01 | 0.98 | ctctttctgGGAAatccct |
| V$BCL6 | POZ domain zinc finger expressed in B-Cells | V$BCL6.01 | 0.835 | gatTTCCcagaaagagc |
| V$HEAT | Heat shock factors | V$HSF1.03 | 0.831 | aagggatttcccAGAAagagctgtt |
| V$STAT | Signal transducer and activator of transcription | V$STAT5.01 | 0.959 | ggatTTCCcagaaagagct |
| V$FKHD | Fork head domain factors | V$HNF3.01 | 0.995 | tgtatgcAAACagctct |
| V$OCT1 | Octamer binding protein | V$OCT1.02 | 0.958 | tgtATGCaaacagctct |
| V$OCT1 | Octamer binding protein | V$OCT3_4.02 | 0.899 | tgtttGCATacatggtg |
| V$SRFF | Serum response element binding factor | V$SRF.02 | 0.845 | gtttgCATAcatggtggcc |
| V$RUSH | SWI/SNF related nucleophosphoproteins with a RING finger DNA binding motif | V$SMARCA3.01 | 0.974 | caCCATgtatg |
| V$SMAD | Vertebrate SMAD family of transcription factors | V$SMAD3.01 | 0.994 | tcaGTCTgggc |
| V$SORY | SOX/SRY-sex/testis determinig and related HMG box factors | V$HBP1.01 | 0.863 | ccagactgACTGagatctgcataga |
| V$GATA | GATA binding factors | V$GATA3.02 | 0.916 | tgcAGATctcagt |
| V$OCT1 | Octamer binding protein | V$POU2F3.01 | 0.881 | tctATGCagatctcagt |
| V$DICE | Downstream Immunoglobulin Control Element, critical for B cell activity and specificity | V$DICE.01 | 0.808 | tgagATCTgcataga |
| V$OCT1 | Octamer binding protein | V$OCT3_4.02 | 0.928 | gatctGCATagagacaa |
| V$SORY | SOX/SRY-sex/testis determinig and related HMG box factors | V$SOX9.03 | 0.774 | tagagACAAgggcgcaatagcagtg |
| V$E2FF | E2F-myc activator/cell cycle regulator | V$E2F1_DP1.01 | 0.818 | caagGGCGcaatagcag |
| V$AIRE | Autoimmune regulatory element binding factor | V$AIRE.01 | 0.864 | cagtgttttggaaactgTGGTaggaag |
| V$HAML | Human acute myelogenous leukemia factors | V$AML1.01 | 0.939 | aactGTGGtaggaag |
| V$ETSF | Human and murine ETS1 factors | V$ELF2.01 | 0.903 | ctgtggtaGGAAggaggcagt |
| V$CTCF | CTCF and BORIS gene family, transcriptional regulators with 11 highly conserved zinc finger domains | V$CTCF.02 | 0.705 | aactgtggtaggaaggaGGCAgtgagt |
| V$CARE | Calcium-response elements | V$CARF.01 | 0.925 | ggaagGAGGca |
| V$AP1R | MAF and AP1 related factors | V$NFE2.01 | 0.889 | aaaccCTGActcactgcctcc |
| V$CREB | cAMP-responsive element binding proteins | V$TAXCREB.02 | 0.742 | aaacccTGACtcactgcctcc |
| V$AP1F | AP1, Activating protein 1 | V$AP1.01 | 0.991 | cagtgAGTCaggg |
| V$AP1F | AP1, Activating protein 1 | V$AP1.01 | 0.945 | ccctgACTCactg |
| V$AP1R | MAF and AP1 related factors | V$BACH2.01 | 0.98 | aggcagTGAGtcagggtttac |
| V$CSEN | Calsenilin, presenilin binding protein, EF hand transcription factor | V$DREAM.01 | 0.974 | gaGTCAgggtt |
| V$IRFF | Interferon regulatory factors | V$IRF3.01 | 0.86 | ctcagaaaaaGATAttgctca |
| V$CARE | Calcium-response elements | V$CARF.01 | 0.923 | acaggGAGGct |
| V$STAT | Signal transducer and activator of transcription | V$STAT6.01 | 0.879 | agacTTCGtccgaagcctc |
| V$STAT | Signal transducer and activator of transcription | V$STAT6.01 | 0.862 | aggcTTCGgacgaagtctg |
| V$IRXF | Iroquois homeobox transcription factors | V$IRX2.01 | 0.86 | catgCATGtacag |
| V$IRXF | Iroquois homeobox transcription factors | V$IRX2.01 | 0.861 | tgtaCATGcatgt |
| V$STAF | Selenocysteine tRNA activating factor | V$STAF.01 | 0.771 | cccaCCCCacatgcatgtacaga |
| V$HAND | Twist subfamily of class B bHLH transcription factors | V$TWIST.01 | 0.944 | gtacatgCATGtggggtgggg |
| V$GCMF | Chorion-specific transcription factors with a GCM DNA binding domain | V$GCM1.01 | 0.868 | cccacCCCAcatgca |
| V$SREB | Sterol regulatory element binding proteins | V$SREBP.02 | 0.838 | accCCACcccacatg |
| V$KLFS | Krueppel like transcription factors | V$BKLF.01 | 0.975 | catgtgGGGTggggttg |
| V$SP1F | GC-Box factors SP1/GC | V$GC.01 | 0.917 | atgtggGGTGgggttgg |
| V$MAZF | Myc associated zinc fingers | V$MAZR.01 | 0.907 | tggggtGGGGttg |
| V$ZF02 | C2H2 zinc finger transcription factors 2 | V$ZF9.01 | 0.882 | tctttccaaccCCACcccacatg |
| V$ZF02 | C2H2 zinc finger transcription factors 2 | V$ZKSCAN3.01 | 1 | tgtctttccaaCCCCaccccaca |
| V$CLOX | CLOX and CLOX homology (CDP) factors | V$CDP.02 | 0.95 | ataggcCAATctgtctttc |
| V$CAAT | CCAAT binding factors | V$NFY.04 | 0.942 | taggCCAAtctgtct |
| V$YBXF | Y-box binding transcription factors, multifunctional proteins involved in transcriptional and translational regulation, mRNA splicing, DNA replication and repair | V$YB1.01 | 0.979 | cagatTGGCctat |
| V$RXRF | RXR heterodimer binding sites | V$VDR_RXR.05 | 0.83 | cttcGAAGttcccataggccaatct |
| V$RBPF | RBPJ - kappa | V$RBPJK.02 | 0.95 | cctaTGGGaactt |
| V$IKRS | Ikaros zinc finger family | V$IK1.01 | 0.928 | ctatGGGAacttc |
| V$BCL6 | POZ domain zinc finger expressed in B-Cells | V$BCL6.03 | 0.82 | ttcttctTCGAagttcc |
| V$STAT | Signal transducer and activator of transcription | V$STAT5.01 | 0.935 | tttcTTCTtcgaagttccc |
| V$STAT | Signal transducer and activator of transcription | V$STAT5.01 | 0.957 | gaacTTCGaagaagaaagt |
| V$IRFF | Interferon regulatory factors | V$IRF7.01 | 0.894 | cttcGAAGaagaaagtcgggg |
| V$PAX6 | PAX-4/PAX-6 paired domain binding sites | V$PAX6.04 | 0.859 | tctCCCCgactttcttctt |
| V$GATA | GATA binding factors | V$GATA3.02 | 0.925 | gagAGATgtgagt |
| V$AP1F | AP1, Activating protein 1 | V$AP1.02 | 0.881 | atgtGAGTgaagg |
| V$RXRF | RXR heterodimer binding sites | V$VDR_RXR.05 | 0.818 | gagaGATGtgagtgaaggaaaggtg |
| V$RXRF | RXR heterodimer binding sites | V$VDR_RXR.06 | 0.765 | atgtgagtgaaggaaAGGTggggaa |
| V$PRDF | Positive regulatory domain I binding factor | V$BLIMP1.01 | 0.817 | gtgaagGAAAggtggggaa |
| V$BARB | Barbiturate-inducible element box from pro+eukaryotic genes | V$BARBIE.01 | 0.881 | aaggAAAGgtgggga |
| V$MZF1 | Myeloid zinc finger 1 factors | V$MZF1.01 | 1 | gtGGGGaaacc |
| V$ZF02 | C2H2 zinc finger transcription factors 2 | V$ZKSCAN3.01 | 1 | ccagtgggtttCCCCacctttcc |
| V$NFKB | Nuclear factor kappa B/c-rel | V$NFKAPPAB50.01 | 0.922 | gtgGGGAaacccact |
| V$PAX6 | PAX-4/PAX-6 paired domain binding sites | V$PAX6.04 | 0.852 | attTCCCcagtgggtttcc |
| V$NOLF | Neuron-specific olfactory factor | V$EBF1.01 | 0.926 | atcattTCCCcagtgggtttccc |
| V$IKRS | Ikaros zinc finger family | V$IK2.01 | 0.981 | ctttGGGAtcatt |
| V$SRFF | Serum response element binding factor | V$SRF.03 | 0.79 | aagaccctctTTGGgatca |
| V$SRFF | Serum response element binding factor | V$SRF.02 | 0.863 | gatccCAAAgagggtcttt |
| V$SORY | SOX/SRY-sex/testis determinig and related HMG box factors | V$SOX9.03 | 0.805 | tgatcCCAAagagggtctttcaggg |
| V$ETSF | Human and murine ETS1 factors | V$ETS2.01 | 0.842 | tcagggcAGGAaaagctgtca |
| V$HOXH | HOX - MEIS1 heterodimers | V$MEIS1A_HOXA9.01 | 0.846 | TGACagcttttcctg |
| V$TALE | TALE homeodomain class recognizing TG motifs | V$TGIF.01 | 1 | gaaaagctGTCAaaaca |
| V$EVI1 | EVI1-myleoid transforming protein | V$EVI1.07 | 0.903 | aacagAAGAagagctcc |
| V$SORY | SOX/SRY-sex/testis determinig and related HMG box factors | V$SOX9.03 | 0.81 | agtggACACaggagctcttcttctg |
| V$BNCF | Basonuclein rDNA transcription factor (PolI) | V$BNC.01 | 0.867 | agagctcctgTGTCcactg |
| V$LEFF | LEF1/TCF | V$LEF1.01 | 0.889 | ctgagagCAAAgggaca |
| V$PTF1 | Pancreas transcription factor 1, heterotrimeric transcription factor | V$PTF1.01 | 0.8 | cacaGCTGgtaaatgtccctt |
| V$ABDB | Abdominal-B type homeodomain transcription factors | V$HOXB9.01 | 0.908 | acagctggTAAAtgtcc |
| V$HNF1 | Hepatic Nuclear Factor 1 | V$HMBOX.01 | 0.835 | cacagctgGTAAatgtc |
| V$AP4R | AP4 and related proteins | V$AP4.02 | 0.962 | ccacacAGCTggtaaat |
| V$HAND | Twist subfamily of class B bHLH transcription factors | V$TAL1ALPHAE47.01 | 0.882 | ttccacaCAGCtggtaaatgt |
| V$MYOD | Myoblast determining factors | V$MYOGENIN.02 | 0.92 | atttacCAGCtgtgtgg |
| V$BRAC | Brachyury gene, mesoderm developmental factor | V$BRACH.01 | 0.681 | tttccacacAGCTggtaaatg |
| V$ZICF | Members of ZIC-family, zinc finger protein of the cerebellum | V$ZIC3.01 | 0.908 | ccacaCAGCtggtaa |
| V$NFAT | Nuclear factor of activated T-cells | V$NFAT.01 | 0.96 | tgtgtGGAAagcccatggg |
| V$NFKB | Nuclear factor kappa B/c-rel | V$CREL.01 | 0.989 | catgggctTTCCaca |
| V$NFAT | Nuclear factor of activated T-cells | V$NFAT5.01 | 0.85 | tgtGGAAagcccatgggtg |
| V$OAZF | Olfactory associated zinc finger protein | V$ROAZ.01 | 0.737 | gaACACccatgggcttt |
| V$GLIF | GLI zinc finger family | V$GLI2.01 | 0.885 | atgaACACccatggg |
| V$EVI1 | EVI1-myleoid transforming protein | V$EVI1.07 | 0.956 | aacacAAGAtgaacacc |
| V$EVI1 | EVI1-myleoid transforming protein | V$EVI1.04 | 0.757 | atgacaacacaaGATGa |
| V$P53F | p53 tumor suppressor | V$P53.06 | 0.771 | ttcatCTTGtgttgtcatgtttc |
| V$BPTF | Bromodomain and PHD domain transcription factors | V$FAC1.01 | 0.981 | atgacAACAca |
| V$NFAT | Nuclear factor of activated T-cells | V$NFAT5.02 | 0.881 | attGGAAacatgacaacac |
| V$NF1F | Nuclear factor 1 | V$NF1.01 | 0.847 | gtgTTGTcatgtttccaattt |
| V$OCT1 | Octamer binding protein | V$OCT1.06 | 0.886 | gaaatgaaAATTggaaa |
| V$PRDF | Positive regulatory domain I binding factor | V$BLIMP1.01 | 0.845 | agaaatGAAAattggaaac |
| V$IRFF | Interferon regulatory factors | V$IRF7.01 | 0.88 | aaaaGAAAtgaaaattggaaa |
| O$INRE | Core promoter initiator elements | O$DINR.01 | 0.967 | ttTCATttctt |
| V$ETSF | Human and murine ETS1 factors | V$SPI1_PU1.01 | 0.83 | aagaaaaaAGAAatgaaaatt |
| V$EVI1 | EVI1-myleoid transforming protein | V$EVI1.01 | 0.725 | gaaaaAAGAaatgaaaa |
| V$IRFF | Interferon regulatory factors | V$IRF4.01 | 0.951 | aaaagaaaaaGAAAaaagaaa |
| V$CIZF | CAS interating zinc finger protein | V$NMP4.01 | 0.97 | agAAAAagaaa |
| V$RXRF | RXR heterodimer binding sites | V$VDR_RXR.04 | 0.81 | gttgatgGGTTaaaaagaaaaagaa |
| V$FKHD | Fork head domain factors | V$XFD3.01 | 0.829 | gatgggttAAAAagaaa |
| V$HNF1 | Hepatic Nuclear Factor 1 | V$HNF1.04 | 0.845 | gttgatggGTTAaaaag |
| V$CAAT | CCAAT binding factors | V$NFY.03 | 0.831 | ttaaCCCAtcaacac |
| V$PAX6 | PAX-4/PAX-6 paired domain binding sites | V$PAX6.03 | 0.784 | ttttaACCCatcaacacat |
| V$EBOX | E-box binding factors | V$MYCMAX.02 | 0.927 | tcaacaCATGtga |
| V$HAND | Twist subfamily of class B bHLH transcription factors | V$MESP1_2.01 | 0.919 | ccatcaaCACAtgtgaaatgg |
| V$IRXF | Iroquois homeobox transcription factors | V$IRX5.01 | 0.895 | ttcaCATGtgttg |
| V$IRXF | Iroquois homeobox transcription factors | V$IRX5.01 | 0.903 | aacaCATGtgaaa |
| V$MITF | Microphthalmia transcription factor | V$MIT.01 | 0.935 | caacaCATGtgaaat |
| V$FAST | FAST-1 SMAD interacting proteins | V$FAST1.02 | 0.813 | acacaTGTGaaatggtt |
| V$PRDF | Positive regulatory domain I binding factor | V$BLIMP1.01 | 0.818 | acatgtGAAAtggttgtgg |
| V$HAML | Human acute myelogenous leukemia factors | V$AML3.01 | 0.864 | ggttGTGGttcagag |
| V$RXRF | RXR heterodimer binding sites | V$PXR_RXR.01 | 0.838 | gttgtgGTTCagagcagtgctgttt |
| V$PARF | PAR/bZIP family | V$HLF.01 | 0.847 | gctgttttgGTAActtt |
| V$SNAP | snRNA-activating protein complex | V$PSE.02 | 0.74 | tatacCCTAaagttaccaa |
| V$NKXH | NKX homeodomain factors | V$NKX31.01 | 0.943 | gacagtAAGTataccctaa |
| V$GREF | Glucocorticoid responsive and related elements | V$ARE.02 | 0.906 | ggtatacttactGTCCgag |
| V$CAAT | CCAAT binding factors | V$NFY.03 | 0.817 | taaaCCAAacatagc |
| V$STAT | Signal transducer and activator of transcription | V$STAT3.02 | 0.97 | gagtTTCCaggtatgctat |
| V$BCL6 | POZ domain zinc finger expressed in B-Cells | V$BCL6.03 | 0.83 | gcataccTGGAaactct |
| V$PAX6 | PAX-4/PAX-6 paired domain binding sites | V$PAX6.02 | 0.877 | gaagagtttCCAGgtatgc |
| V$NFAT | Nuclear factor of activated T-cells | V$NFAT5.02 | 0.893 | cctGGAAactcttctttat |
| V$EVI1 | EVI1-myleoid transforming protein | V$EVI1.07 | 0.916 | aaataAAGAagagtttc |
| V$SORY | SOX/SRY-sex/testis determinig and related HMG box factors | V$HMGA.01 | 0.889 | ggttgctgataAATAaagaagagtt |
| V$HOXC | HOX - PBX complexes | V$PBX_HOXA9.01 | 0.794 | ttctTTATttatcagca |
| V$ABDB | Abdominal-B type homeodomain transcription factors | V$HOXC13.02 | 0.832 | gttgctgaTAAAtaaag |
| V$GATA | GATA binding factors | V$GATA.01 | 0.979 | tgctGATAaataa |
| V$MEF3 | MEF3 binding sites | V$SIX.01 | 0.918 | tatTCTGgttgct |
| V$NRSF | Neuron-restrictive silencer factor | V$NRSF.01 | 0.726 | ttatcAGCAaccagaatagaagctgagtcac |
| V$AP1R | MAF and AP1 related factors | V$TCF11MAFG.01 | 0.848 | atcctgTGACtcagcttctat |
| V$AP1F | AP1, Activating protein 1 | V$AP1.01 | 0.968 | agctgAGTCacag |
| V$AP1F | AP1, Activating protein 1 | V$AP1.01 | 0.968 | ctgtgACTCagct |
| V$AP1R | MAF and AP1 related factors | V$NFE2.01 | 0.989 | agaagCTGAgtcacaggatga |
| V$PBXC | PBX1 - MEIS1 complexes | V$PBX1_MEIS1.03 | 0.789 | aagctgagTCACaggat |
| V$RORA | v-ERB and RAR-related orphan receptor alpha | V$REV-ERBA.02 | 0.817 | tagaagctgaGTCAcaggatgac |
| V$ETSF | Human and murine ETS1 factors | V$PDEF.01 | 0.948 | gagtcacaGGATgacaccaac |
| V$PAX5 | PAX-5 B-cell-specific activator protein | V$PAX5.03 | 0.806 | ctgagTCACaggatgacaccaacacatct |
| V$SIXF | Sine oculis (SIX) homeodomain factors | V$SIX4.01 | 0.987 | gtgttgGTGTcatcc |
| V$EGRF | EGR/nerve growth factor induced protein C & related factors | V$EGR2.01 | 0.821 | agatGTGTtggtgtcat |
| V$HAND | Twist subfamily of class B bHLH transcription factors | V$MESP1_2.01 | 0.93 | cagaaacCAGAtgtgttggtg |
| V$RP58 | RP58 (ZFP238) zinc finger protein | V$RP58.01 | 0.925 | aacaCATCtggtt |
| V$MYOD | Myoblast determining factors | V$TCFE2A.02 | 0.948 | agaaaccaGATGtgttg |
| V$MEF3 | MEF3 binding sites | V$SIX.01 | 0.91 | acaTCTGgtttct |
| V$PTF1 | Pancreas transcription factor 1, heterotrimeric transcription factor | V$PTF1.01 | 0.776 | cacaTCTGgtttctgtctcat |
| V$IRFF | Interferon regulatory factors | V$IRF4.03 | 0.876 | gaatgagacaGAAAccagatg |
| V$SORY | SOX/SRY-sex/testis determinig and related HMG box factors | V$HBP1.01 | 0.901 | taaggaagAATGagacagaaaccag |
| V$TEAF | TEA/ATTS DNA binding domain factors | V$TEAD.01 | 0.902 | tctCATTcttcct |
| V$ETSF | Human and murine ETS1 factors | V$SPIB.01 | 0.918 | ggaaataaGGAAgaatgagac |
| V$ETSF | Human and murine ETS1 factors | V$ELF3.01 | 0.943 | aacacccaGGAAataaggaag |
