# Supplementary material for: Cell Specific CD44 Expression in Breast Cancer Requires the Interaction of AP-1 and NFκB with a Novel cis-Element
Source: PLoS One. 2012 Nov 30;7(11):e50867. doi: 10.1371/journal.pone.0050867 (PMC3511339; doi:10.1371/journal.pone.0050867)
Supplement: Table S7 — Predicted transcription factor binding sites from human CD44CR1. (DOC) [file pone.0050867.s011.doc]

**Table S7. Predicted transcription factor binding sites from human CD44CR1.**

| **Matrix Family** | **Detailed Family Information** | **Matrix** | **Matrix sim.** | **Sequence** |
| --- | --- | --- | --- | --- |
| V$GABF | GA-boxes | V$GAGA.01 | 0.79 | tagagAGTGagagatcgaaagatga |
| V$PRDF | Positive regulatory domain I binding factor | V$BLIMP1.01 | 0.817 | gagatcGAAAgatgaggag |
| V$BARB | Barbiturate-inducible element box from pro+eukaryotic genes | V$BARBIE.01 | 0.898 | atcgAAAGatgagga |
| V$EVI1 | EVI1-myleoid transforming protein | V$MEL1.02 | 0.994 | atcgaaaGATGaggagg |
| V$ZF07 | C2H2 zinc finger transcription factors 7 | V$ZNF263.01 | 0.938 | attccCTCCtcatct |
| V$SPZ1 | Testis-specific bHLH-Zip transcription factors | V$SPZ1.01 | 0.957 | aGGAGggaatc |
| V$NFKB | Nuclear factor kappa B/c-rel | V$CREL.01 | 0.939 | atgggtgaTTCCctc |
| V$NFKB | Nuclear factor kappa B/c-rel | V$NFKAPPAB.01 | 0.932 | gaGGGAatcacccat |
| V$HOXC | HOX - PBX complexes | V$PBX1.01 | 0.785 | ctcttGATGggtgattc |
| V$FKHD | Fork head domain factors | V$FREAC7.01 | 0.986 | tgtacaTAAAcagactc |
| V$FKHD | Fork head domain factors | V$FREAC3.01 | 0.876 | acgatGTACataaacag |
| V$BNCF | Basonuclein rDNA transcription factor (PolI) | V$BNC.01 | 0.872 | ggggccacgaTGTAcataa |
| O$TF2D | General transcription factor IID, GTF2D | O$INR_DPE.01 | 0.728 | tcactcttgtctctatgcagatctcggtagGTCGgggcc |
| V$OCT1 | Octamer binding protein | V$POU2F3.01 | 0.881 | tctATGCagatctcggt |
| V$OCT1 | Octamer binding protein | V$OCT3_4.02 | 0.928 | gatctGCATagagacaa |
| V$PAX6 | PAX-4/PAX-6 paired domain binding sites | V$PAX6.04 | 0.84 | attTCACtcttgtctctat |
| V$P53F | p53 tumor suppressor | V$P53.05 | 0.786 | gagaCAAGagtgaaataagactg |
| V$PRDF | Positive regulatory domain I binding factor | V$PRDM1.01 | 0.825 | aagagtGAAAtaagactgt |
| V$BPTF | Bromodomain and PHD domain transcription factors | V$FAC1.01 | 0.961 | tccaaAACAca |
| V$AIRE | Autoimmune regulatory element binding factor | V$AIRE.01 | 0.893 | ctgtgttttggaaactgTGGTaagaag |
| V$CEBP | Ccaat/Enhancer Binding Protein | V$CEBP.02 | 0.922 | aaactgtgGTAAgaa |
| V$HAML | Human acute myelogenous leukemia factors | V$AML1.01 | 0.978 | aactGTGGtaagaag |
| V$RXRF | RXR heterodimer binding sites | V$VDR_RXR.06 | 0.78 | actgtggtaagaaggAGGCaatgag |
| V$CARE | Calcium-response elements | V$CARF.01 | 0.974 | agaagGAGGca |
| V$CAAT | CCAAT binding factors | V$NFY.03 | 0.829 | ggagGCAAtgagtca |
| V$NR2F | Nuclear receptor subfamily 2 factors | V$TR2_TR4.01 | 0.776 | agaaggaggcaatgAGTCagtgctt |
| V$AP1R | MAF and AP1 related factors | V$NFE2.01 | 0.909 | aagcaCTGActcattgcctcc |
| V$CREB | cAMP-responsive element binding proteins | V$TAXCREB.02 | 0.776 | aagcacTGACtcattgcctcc |
| V$AP1F | AP1, Activating protein 1 | V$AP1.01 | 1 | caatgAGTCagtg |
| V$AP1F | AP1, Activating protein 1 | V$AP1.03 | 0.952 | cacTGACtcattg |
| V$AP1R | MAF and AP1 related factors | V$BACH2.01 | 0.967 | aggcaaTGAGtcagtgcttac |
| V$INSM | Insulinoma associated factors | V$INSM1.01 | 0.905 | tttctGGGGatgg |
| V$IRFF | Interferon regulatory factors | V$IRF3.01 | 0.86 | cccagaaaaaGATAttgctca |
| V$AP1R | MAF and AP1 related factors | V$MAFK.01 | 0.85 | aagatatTGCTcactggggag |
| V$NOLF | Neuron-specific olfactory factor | V$EBF1.01 | 0.933 | aaagacTCCCcagtgagcaatat |
| V$ZF02 | C2H2 zinc finger transcription factors 2 | V$ZNF300.01 | 0.994 | tgccccaaagactCCCCagtgag |
| V$RREB | Ras-responsive element binding protein | V$RREB1.01 | 0.853 | cCCCAaagactcccc |
| V$E2FF | E2F-myc activator/cell cycle regulator | V$E2F.02 | 0.849 | actttgcccCAAAgact |
| V$NR2F | Nuclear receptor subfamily 2 factors | V$HNF4.01 | 0.871 | gtctttggggCAAAgtctgtacatg |
| V$P53F | p53 tumor suppressor | V$P53.05 | 0.808 | ggggCAAAgtctgtacatgtatc |
| V$IRXF | Iroquois homeobox transcription factors | V$IRX5.01 | 0.993 | gataCATGtacag |
| V$IRXF | Iroquois homeobox transcription factors | V$IRX2.01 | 0.987 | tgtaCATGtatct |
| V$SP1F | GC-Box factors SP1/GC | V$GC.01 | 0.904 | tctgggGGTGgagttgg |
| V$ZF02 | C2H2 zinc finger transcription factors 2 | V$ZBP89.01 | 0.959 | ctttccaactccaCCCCcagata |
| V$CLOX | CLOX and CLOX homology (CDP) factors | V$CDP.02 | 0.95 | atatgcCAATctgtctttc |
| V$CAAT | CCAAT binding factors | V$NFY.04 | 0.93 | tatgCCAAtctgtct |
| V$OCT1 | Octamer binding protein | V$OCT1.01 | 0.934 | caTATGccaatctgtct |
| V$HICF | Krueppel-like C2H2 zinc finger factors hypermethylated in cancer | V$HIC1.01 | 0.881 | ataTGCCaatctg |
| V$YBXF | Y-box binding transcription factors, multifunctional proteins involved in transcriptional and translational regulation, mRNA splicing, DNA replication and repair | V$YB1.01 | 0.925 | cagatTGGCatat |
| V$HAND | Twist subfamily of class B bHLH transcription factors | V$MESP1_2.01 | 0.951 | aaataccCATAtgccaatctg |
| V$RUSH | SWI/SNF related nucleophosphoproteins with a RING finger DNA binding motif | V$SMARCA3.01 | 0.961 | acCCATatgcc |
| V$ARID | AT rich interactive domain factor | V$MRF2.01 | 0.985 | tccaAATAcccatatgccaat |
| V$GCMF | Chorion-specific transcription factors with a GCM DNA binding domain | V$GCM1.03 | 0.852 | aaataCCCAtatgcc |
| V$HMTB | Human muscle-specific Mt binding site | V$MTBF.01 | 0.979 | gggtATTTg |
| V$STAT | Signal transducer and activator of transcription | V$STAT.01 | 0.882 | gtatttggaGGAAagcagt |
| V$NFAT | Nuclear factor of activated T-cells | V$NFAT.01 | 0.971 | ttggaGGAAagcagtggga |
| V$BARB | Barbiturate-inducible element box from pro+eukaryotic genes | V$BARBIE.01 | 0.882 | gaggAAAGcagtggg |
| V$GABF | GA-boxes | V$GAGA.01 | 0.794 | aaagcAGTGggagagagagagaatg |
| V$GABF | GA-boxes | V$GAGA.01 | 0.818 | cagtgGGAGagagagagaatgtgaa |
| V$GABF | GA-boxes | V$GAGA.01 | 0.838 | gtgggAGAGagagagaatgtgaatg |
| V$GABF | GA-boxes | V$GAGA.01 | 0.847 | gggagAGAGagagaatgtgaatgaa |
| V$GABF | GA-boxes | V$GAGA.01 | 0.794 | gagagAGAGagaatgtgaatgaatg |
| V$IRFF | Interferon regulatory factors | V$IRF7.01 | 0.862 | gagaGAATgtgaatgaatgaa |
| V$OCT1 | Octamer binding protein | V$OCT1.04 | 0.82 | agAATGtgaatgaatga |
| V$HOXF | Paralog hox genes 1-8 from the four hox clusters A, B, C, D | V$NANOG.01 | 0.947 | gaatgtgAATGaatgaaaa |
| V$SORY | SOX/SRY-sex/testis determinig and related HMG box factors | V$HBP1.02 | 0.989 | gaatgtgAATGaatgaaaagtggga |
| V$PBXC | PBX1 - MEIS1 complexes | V$PBX1_MEIS1.01 | 0.742 | tgaaTGAAtgaaaagtg |
| V$SORY | SOX/SRY-sex/testis determinig and related HMG box factors | V$HBP1.01 | 1 | tgtgaatgAATGaaaagtgggaaaa |
| V$HOMF | Homeodomain transcription factors | V$HMX3.01 | 0.922 | tgaatgaaAAGTgggaaaa |
| V$NKXH | NKX homeodomain factors | V$NKX26.01 | 0.836 | aatgaaaAGTGggaaaaca |
| V$E2FF | E2F-myc activator/cell cycle regulator | V$E2F.01 | 0.752 | gaaaagtggGAAAacac |
| V$RBPF | RBPJ - kappa | V$RBPJK.02 | 0.976 | aaagTGGGaaaac |
| V$IKRS | Ikaros zinc finger family | V$IK2.01 | 0.98 | aagtGGGAaaaca |
| V$FKHD | Fork head domain factors | V$ILF1.01 | 1 | agtgggaaAACAcattg |
| V$FAST | FAST-1 SMAD interacting proteins | V$FAST1.02 | 0.816 | cccaaTGTGttttccca |
| V$PAX6 | PAX-4/PAX-6 paired domain binding sites | V$PAX6.04 | 0.887 | tttTCCCcaatgtgttttc |
| V$GCMF | Chorion-specific transcription factors with a GCM DNA binding domain | V$GCM1.03 | 0.867 | gttttCCCCaatgtg |
| V$MZF1 | Myeloid zinc finger 1 factors | V$MZF1.02 | 0.995 | ttGGGGaaaac |
| V$FKHD | Fork head domain factors | V$ILF1.01 | 1 | ttggggaaAACAcatag |
| V$GREF | Glucocorticoid responsive and related elements | V$ARE.01 | 0.847 | tcggtactatgTGTTttcc |
| V$DMRT | DM domain-containing transcription factors | V$DMRT5.01 | 0.812 | cgctttcGGTActatgtgttt |
| V$RU49 | Zinc finger transcription factor RU49, zinc finger proliferation 1 - Zipro1 | V$RU49.01 | 0.991 | tAGTAcc |
| V$PAX5 | PAX-5 B-cell-specific activator protein | V$PAX5.02 | 0.731 | cacatagtaccgaaAGCGggtctctgagg |
| V$IRFF | Interferon regulatory factors | V$IRF4.02 | 0.706 | taccGAAAgcgggtctctgag |
| V$MYOD | Myoblast determining factors | V$MYOD.01 | 0.883 | ctgaGGCAgaaggagcc |
| V$ABDB | Abdominal-B type homeodomain transcription factors | V$HOXB9.02 | 0.906 | aggagccaTCAAagcgg |
| V$LEFF | LEF1/TCF | V$LEF1.02 | 0.971 | gagccatCAAAgcggaa |
| V$ETSF | Human and murine ETS1 factors | V$SPI1_PU1.02 | 0.971 | atcaaagcGGAAgcagaactc |
| V$PAX3 | PAX-3 binding sites | V$PAX3.01 | 0.805 | aTCGAcacggcactgagtt |
| V$OCT1 | Octamer binding protein | V$OCT1.02 | 0.851 | cctATGCaatcttcaaa |
| V$CHOP | C/EBP homologous protein (CHOP) | V$CHOP.01 | 0.924 | ctatGCAAtcttc |
| V$OCT1 | Octamer binding protein | V$POU3F3.01 | 0.811 | agattGCATagggaacc |
| V$XBBF | X-box binding factors | V$RFX1.02 | 0.901 | agattgcatagGGAAccca |
| V$SORY | SOX/SRY-sex/testis determinig and related HMG box factors | V$HBP1.01 | 0.866 | atgaaatgAATGtctctgggttccc |
| V$HOXF | Paralog hox genes 1-8 from the four hox clusters A, B, C, D | V$NANOG.01 | 0.949 | tgaaatgAATGtctctggg |
| V$BRNF | Brn POU domain factors | V$BRN2.03 | 0.966 | gagacattcATTTcatttt |
| V$IRFF | Interferon regulatory factors | V$IRF1.01 | 0.885 | tgaaaaaaatGAAAtgaatgt |
| V$OCT1 | Octamer binding protein | V$OCT1.05 | 0.916 | ttCATTtcatttttttc |
| O$INRE | Core promoter initiator elements | O$DINR.01 | 0.949 | ttTCATttttt |
| V$OCT1 | Octamer binding protein | V$OCT1.04 | 0.814 | agCATGaaaaaaatgaa |
| V$PAX8 | PAX-2/5/8 binding sites | V$PAX2.02 | 0.983 | ttTTCAtgcttctaa |
| V$HOMF | Homeodomain transcription factors | V$MSX.01 | 0.989 | atgcttcTAATtttaattt |
| V$MEF2 | MEF2, myocyte-specific enhancer binding factor | V$SL1.01 | 0.867 | catgcttCTAAttttaatttctt |
| V$ARID | AT rich interactive domain factor | V$BRIGHT.01 | 0.952 | aagaaATTAaaattagaagca |
| V$HOMF | Homeodomain transcription factors | V$HHEX.01 | 0.98 | gcttctaatttTAATttct |
| V$LHXF | Lim homeodomain factors | V$LHX3.02 | 0.84 | atgcttctaatttTAATttcttt |
| V$DLXF | Distal-less homeodomain transcription factors | V$DLX3.01 | 0.925 | ttctaatttTAATttcttt |
| V$IRFF | Interferon regulatory factors | V$IRF7.01 | 0.892 | aaaaGAAAttaaaattagaag |
| V$HBOX | Homeobox transcription factors | V$EN1.01 | 0.775 | aaaagaaaTTAAaattaga |
| V$HOXF | Paralog hox genes 1-8 from the four hox clusters A, B, C, D | V$HOXB8.01 | 0.889 | aaaagaaATTAaaattaga |
| V$OCT1 | Octamer binding protein | V$OCT1.06 | 0.838 | ctaattttAATTtcttt |
| V$ABDB | Abdominal-B type homeodomain transcription factors | V$HOXB9.02 | 0.88 | aaaagaaaTTAAaatta |
| V$ARID | AT rich interactive domain factor | V$JARID2.01 | 0.89 | tctaatTTTAatttcttttca |
| V$BRNF | Brn POU domain factors | V$BRN3.02 | 0.906 | ctaatttTAATttcttttc |
| V$HOMF | Homeodomain transcription factors | V$MSX.01 | 0.989 | ctaatttTAATttcttttc |
| V$NKX6 | NK6 homeobox transcription factors | V$NKX61.01 | 0.961 | aattTTAAtttcttt |
| V$LHXF | Lim homeodomain factors | V$ISL2.01 | 0.897 | gctgaaaagaaATTAaaattaga |
| V$CART | Cart-1 (cartilage homeoprotein 1) | V$MIXL1.01 | 0.826 | aatttTAATttcttttcagct |
| V$IRFF | Interferon regulatory factors | V$IRF4.01 | 0.951 | gagctgaaaaGAAAttaaaat |
| V$PAX2 | PAX-2 binding sites | V$PAX2.01 | 0.797 | tgagctgaaaagaaattAAAAtt |
| V$GATA | GATA binding factors | V$GATA5.01 | 0.839 | agctGAAAagaaa |
| V$EVI1 | EVI1-myleoid transforming protein | V$EVI1.04 | 0.764 | ttgagctgaaaaGAAAt |
| V$CREB | cAMP-responsive element binding proteins | V$XBP1.01 | 0.906 | attatttcACGTgttgagctg |
| V$EBOX | E-box binding factors | V$MYCMAX.02 | 0.984 | tcaacaCGTGaaa |
| V$HESF | Vertebrate homologues of enhancer of split complex | V$BHLHB2.01 | 0.914 | atttcACGTgttgag |
| V$HIFF | Hypoxia inducible factor, bHLH/PAS protein family | V$ARNT.01 | 0.942 | gctcaacaCGTGaaata |
| V$EBOX | E-box binding factors | V$USF.01 | 0.981 | atttCACGtgttg |
| V$HESF | Vertebrate homologues of enhancer of split complex | V$DEC2.01 | 0.973 | tcaacaCGTGaaata |
| V$HIFF | Hypoxia inducible factor, bHLH/PAS protein family | V$ARNT.01 | 0.966 | ttatttcaCGTGttgag |
| V$CHRE | Carbohydrate response elements, consist of two E box motifs separated by 5 bp | V$CHREBP_MLX.01 | 0.829 | CACGtgaaataattgtg |
| V$HBOX | Homeobox transcription factors | V$GSH1.01 | 0.917 | acacgtgaaaTAATtgtgg |
| V$SORY | SOX/SRY-sex/testis determinig and related HMG box factors | V$SOX13.01 | 0.863 | aaaccACAAttatttcacgtgttga |
| V$DLXF | Distal-less homeodomain transcription factors | V$DLX2.01 | 0.932 | cacgtgaaatAATTgtggt |
| V$HOXF | Paralog hox genes 1-8 from the four hox clusters A, B, C, D | V$HOXD3.01 | 0.851 | aaccacAATTatttcacgt |
| V$HOMF | Homeodomain transcription factors | V$BARX2.01 | 0.984 | cgtgaaaTAATtgtggttt |
| V$NKX1 | NK1 homeobox transcription factors | V$NKX12.01 | 0.882 | aaccacAATTatttcac |
| V$BCDF | Bicoid-like homeodomain transcription factors | V$PCE1.01 | 0.891 | tgaaaTAATtgtggttt |
| V$HBOX | Homeobox transcription factors | V$EN2.01 | 0.863 | gtgaaatAATTgtggttta |
| V$FKHD | Fork head domain factors | V$FHXB.01 | 0.832 | taaaccACAAttatttc |
| V$HOXF | Paralog hox genes 1-8 from the four hox clusters A, B, C, D | V$HOX1-3.01 | 0.891 | tgaaaTAATtgtggtttaa |
| V$NKXH | NKX homeodomain factors | V$NKX25.02 | 0.883 | tgaaaTAATtgtggtttaa |
| V$CART | Cart-1 (cartilage homeoprotein 1) | V$S8.01 | 0.995 | tgaaaTAATtgtggtttaaag |
| V$DLXF | Distal-less homeodomain transcription factors | V$DLX2.01 | 0.933 | tttaaaccacAATTatttc |
| V$HAML | Human acute myelogenous leukemia factors | V$AML3.01 | 0.9 | aattGTGGtttaaag |
| O$VTBP | Vertebrate TATA binding protein factor | O$MTATA.01 | 0.871 | tgcttTAAAccacaatt |
| V$IRFF | Interferon regulatory factors | V$IRF4.03 | 0.976 | taaggttaccGAAActacatt |
| V$PARF | PAR/bZIP family | V$HLF.01 | 0.847 | gtagtttcgGTAAcctt |
| V$HOMF | Homeodomain transcription factors | V$TLX1.01 | 0.844 | gtagtttCGGTaaccttag |
| V$SNAP | snRNA-activating protein complex | V$PSE.02 | 0.751 | atagcCCTAaggttaccga |
| V$MEF2 | MEF2, myocyte-specific enhancer binding factor | V$SL1.01 | 0.872 | cttagggCTATacttatgtccaa |
| V$NKXH | NKX homeodomain factors | V$NKX31.01 | 0.95 | ggacatAAGTatagcccta |
| V$RUSH | SWI/SNF related nucleophosphoproteins with a RING finger DNA binding motif | V$SMARCA3.02 | 0.993 | ctatACTTatg |
| V$MYT1 | MYT1 C2HC zinc finger protein | V$MYT1.02 | 0.88 | tccAAGTttaggg |
| V$HNF1 | Hepatic Nuclear Factor 1 | V$HNF1.04 | 0.889 | tatgtttgGTTAagccc |
| V$CAAT | CCAAT binding factors | V$NFY.03 | 0.824 | ttaaCCAAacatagc |
| V$FKHD | Fork head domain factors | V$FREAC4.01 | 0.788 | cttaaccaAACAtagca |
| V$STAT | Signal transducer and activator of transcription | V$STAT3.02 | 0.97 | ccatTTCCaggtatgctat |
| V$BCL6 | POZ domain zinc finger expressed in B-Cells | V$BCL6.03 | 0.827 | gcataccTGGAaatggt |
| V$STAT | Signal transducer and activator of transcription | V$STAT1.02 | 0.85 | agcatacctGGAAatggtc |
| V$PAX6 | PAX-4/PAX-6 paired domain binding sites | V$PAX6.02 | 0.892 | ggaccatttCCAGgtatgc |
| V$ETSF | Human and murine ETS1 factors | V$ELF3.01 | 0.916 | gcatacctGGAAatggtcctt |
| V$MOKF | Mouse Krueppel like factor | V$MOK2.02 | 0.984 | acctggaaatggtCCTTtatt |
| V$PLZF | C2H2 zinc finger protein PLZF | V$PLZF.01 | 0.886 | aaaTAAAggaccatt |
| V$HOXC | HOX - PBX complexes | V$PBX_HOXA9.01 | 0.794 | tcctTTATttatcagta |
| V$GATA | GATA binding factors | V$GATA.01 | 0.979 | tactGATAaataa |
| V$HNF1 | Hepatic Nuclear Factor 1 | V$HNF1.02 | 0.806 | agcTACTgataaataaa |
| V$OSRF | Odd-skipped related factors | V$OSR1.01 | 0.91 | tatcaGTAGctga |
| O$VTBP | Vertebrate TATA binding protein factor | O$ATATA.01 | 0.801 | ttctattTCAGctactg |
| V$IRFF | Interferon regulatory factors | V$IRF7.01 | 0.899 | agctGAAAtagaagctgagtc |
| V$AP1R | MAF and AP1 related factors | V$TCF11MAFG.01 | 0.848 | ctcctgTGACtcagcttctat |
| V$AP1F | AP1, Activating protein 1 | V$AP1.01 | 0.968 | agctgAGTCacag |
| V$AP1F | AP1, Activating protein 1 | V$AP1.01 | 0.968 | ctgtgACTCagct |
| V$AP1R | MAF and AP1 related factors | V$NFE2.01 | 0.989 | agaagCTGAgtcacaggagcg |
| V$PBXC | PBX1 - MEIS1 complexes | V$PBX1_MEIS1.03 | 0.789 | aagctgagTCACaggag |
| V$RORA | v-ERB and RAR-related orphan receptor alpha | V$REV-ERBA.02 | 0.817 | tagaagctgaGTCAcaggagcga |
| V$HAND | Twist subfamily of class B bHLH transcription factors | V$MESP1_2.01 | 0.914 | cagacacCAGAtgtgtttgtg |
| V$RP58 | RP58 (ZFP238) zinc finger protein | V$RP58.01 | 0.925 | aacaCATCtggtg |
| V$MYOD | Myoblast determining factors | V$TCFE2A.02 | 0.948 | agacaccaGATGtgttt |
| V$PTF1 | Pancreas transcription factor 1, heterotrimeric transcription factor | V$PTF1.01 | 0.769 | cacaTCTGgtgtctgtctcat |
| V$SMAD | Vertebrate SMAD family of transcription factors | V$SMAD.01 | 0.989 | ggtGTCTgtct |
| V$CHRE | Carbohydrate response elements, consist of two E box motifs separated by 5 bp | V$CHREBP_MLX.01 | 0.837 | CATGagacagacaccag |
| V$PAX8 | PAX-2/5/8 binding sites | V$PAX2.02 | 0.924 | gtCTCAtgccttcaa |
| V$CHRF | Cell cycle regulators: Cell cycle homology element | V$CHR.01 | 0.946 | gcatTTGAaggca |
| V$HMTB | Human muscle-specific Mt binding site | V$MTBF.01 | 0.922 | tggcATTTg |
| V$AP2F | Activator protein 2 | V$AP2.02 | 0.924 | aatGCCAcagggtgt |
| V$EGRF | EGR/nerve growth factor induced protein C & related factors | V$EGR2.01 | 0.796 | caggGTGTtggcgatct |
